# Insights into the post-translational modification and its emerging role in shaping the tumor microenvironment

**DOI:** 10.1038/s41392-021-00825-8

**Published:** 2021-12-20

**Authors:** Wen Li, Feifei Li, Xia Zhang, Hui-Kuan Lin, Chuan Xu

**Affiliations:** 1 Integrative Cancer Center & Cancer Clinical Research Center, Sichuan Cancer Hospital & Institute, Sichuan Cancer Center, School of Medicine, University of Electronic Science and Technology of China, 610042 Chengdu, P. R. China; 2 Guangxi Collaborative Innovation Center for Biomedicine (Guangxi-ASEAN Collaborative Innovation Center for Major Disease Prevention and Treatment), Guangxi Medical University, 530021 Nanning, Guangxi China; 3 Institute of Pathology and Southwest Cancer Center, Southwest Hospital, Third Military Medical University (Army Medical University), 400038 Chongqing, China; 4 Department of Cancer Biology, Wake Forest Baptist Medical Center, Wake Forest University, Winston Salem, NC 27101 USA

**Keywords:** Cancer microenvironment, Oncogenes

## Abstract

More and more in-depth studies have revealed that the occurrence and development of tumors depend on gene mutation and tumor heterogeneity. The most important manifestation of tumor heterogeneity is the dynamic change of tumor microenvironment (TME) heterogeneity. This depends not only on the tumor cells themselves in the microenvironment where the infiltrating immune cells and matrix together forming an antitumor and/or pro-tumor network. TME has resulted in novel therapeutic interventions as a place beyond tumor beds. The malignant cancer cells, tumor infiltrate immune cells, angiogenic vascular cells, lymphatic endothelial cells, cancer-associated fibroblastic cells, and the released factors including intracellular metabolites, hormonal signals and inflammatory mediators all contribute actively to cancer progression. Protein post-translational modification (PTM) is often regarded as a degradative mechanism in protein destruction or turnover to maintain physiological homeostasis. Advances in quantitative transcriptomics, proteomics, and nuclease-based gene editing are now paving the global ways for exploring PTMs. In this review, we focus on recent developments in the PTM area and speculate on their importance as a critical functional readout for the regulation of TME. A wealth of information has been emerging to prove useful in the search for conventional therapies and the development of global therapeutic strategies.

## Introduction

Tumor is a complex entity composed of multifactors to control its initiation, progression, and treatment response. The self-sufficient growth signals, the insensitive anti-growth signals, the resistance to apoptosis, the limitless replicative potential, the persistent angiogenesis, the capability for invasion and metastasis, the reprogramed cellular metabolism, and the evading immune destruction are known to be major hallmarks of cancer.^1,2^ These physiologic changes during tumor development suggest a conceptual rationale for the individualized precise treatment and provide clues for the exploration of new therapy strategies. The overarching task of cancer research is to clarify the functional crosstalk of tumor cells and the surrounding physical and cellular environment, also known as the tumor microenvironment (TME). TME embodies (1) angiogenic vascular cells (AVCs) that contain the tube-forming endothelial cells and multiple angiogenic factors; (2) infiltrating immune cells (IICs) that contain both myeloid- and lymphoid-lineage cells, mitogenic growth mediators, and proteolytic enzymes; (3) cancer-associated fibroblastic cells (CAFs) that have diverse functions in different organ-specific TME.^2^ The multiple factors in TME and their contributions to cancer hallmarks make TME as a new target for personalized diagnostics and therapeutics.

The abundant vascular system in TME provides nutritional supports for tumor growth and development. IICs including granulocytes, lymphocytes, and macrophages participate in the regulation of local inflammatory response. CAFs help the migration of tumor cells from the primary site to the blood or other metastatic sites and provide channels for angiogenesis of endothelial cells.^3,4^ The molecular composition and spatial heterogeneity of TME match its functions of promoting and inhibiting cancer, and vary dynamically between different tumor types.^4^ Conventional radiotherapy and chemotherapy will reshape the heterogeneity of TME and further affect the follow-up treatment response.^5,6^ The most important regulation pattern of cell–cell interaction in TME is the adaptation and resistance to local immune response, including the dual role of TME in promoting and inhibiting tumors by different lymphocytes crosstalk.^7^ For example, cytotoxic CD8^+^ memory T cells infiltrated in TME are supported by interferon (IFN-γ) and interleukin-2 (IL-2) released by CD4^+^ T-helper cell 1 (Th1), which can kill tumor cells and promote good prognosis by recognizing tumor-specific antigens to activate the immune response.^7,8^ The Th2 cells can promote B-cell response by producing IL-4, IL-5, and IL-13.^9^ In contrast, Th17 cells produce IL-17A, IL-17F, IL-21, and IL-22 to promote tumor growth.^10^ Hypoxic environment in TME can trigger abnormal tumor adaptation and increase its invasiveness and resistance to oxygen-dependent therapy.^11–13^ Therefore, tumor cells themselves affect TME by releasing cytokines and regulatory small molecules, dynamic changing the dialog with immune cells and local microbiota.^14^ Genomic and proteomic changes of tumor cells play important roles in the reaction of nutrition, growth, immunity, and mechanical stress in TME. Among them, a variety of protein post-translational modifications regulate the interaction of internal and external signals in tumorigenesis and cancer development. The modification of key signal pathway-related proteins in different cells of TME may have multiple levels of fine regulation patterns, so as to participate in the proliferation, activation, and metabolic reprogramming of immune cells, and then affect the outcome of tumor immunotherapy.

Protein post-translational modifications (PTMs) introduce structural changes in existing proteins to participate in multiple biological processes. Other than the coding diversity of human genes by the specific alternative mRNA splicing, protein PTM on the side chains or backbones promotes the increased complexity from genome level to proteome level, which is the key of proteome diversity. One of the categories of PTM is the covalent conjunction of some chemical groups to protein side chains through enzyme catalyzation, and the other is the cleavage of a protein backbone through proteases or autocatalytic cleavage at a specific peptide.^15^ PTM enzymes can be divided into three subtypes based on their functional specificity; the “writers” are responsible for adding substrates, the “readers” recognize modified proteins to initiate downstream signaling cascade, and the “erasers” are best known for their role in PTMs removing. One site in the same protein may undergo one or more types of modifications. Similarly, one modulator can perform multiple roles. This crosstalk between PTMs may be positive or negative based on their activation or inhibition function of downstream signals.^16^ PTMs often modulate electrostatic or structural properties of target proteins to change protein–protein interaction.^17^ Some modifications provide a scaffold or docking site for the interaction of kinases and their specific substrates.^18^

With the classification by protein signature, PTM occurs on both histone and nonhistone proteins. Histone modifications are also called histone marks, which play a crucial role in chromatin organization and cellular function such as transcription. These marks are often added on lysine residues with multiple types like acetylation, methylation, propionylation, butyrylation, crotonylation, 2-hydroxyisobutyrylation, malonylation, and succinylation.^19^ The middle-down proteomics workflow using high-resolution mass spectrometry (MS) is helpful to quantify histone H3 marks.^20^ Some databases like http://dbPTM.mbc.nctu.edu.tw/ and http://ptmcode.embl.de are developed to validate novel PTM type, predict the potential PTM targets, analysis PTM crosstalk, and assess PTM-associated diseases.^21,22^

A variety of PTMs have greatly expanded the functional field of proteins, which is particularly important in the immune recognition of tumor therapy. In particular, many peptides presented to T cells through histocompatibility complex are post-translational modified. The regulation of this process may affect the selection of therapeutic targets and the effect of the immune response. In addition to the well-characterized protein PTMs like phosphorylation, methylation, ubiquitination, and sumoylation, other highly prevalent yet less-studied PTMs have attracted considerable attentions in recent years. Although more than 300 PTMs have been identified due to the development of technology, only a few have functional research results at the proteome level.^23^ Comprehensive and systematic PTM analysis is very challenging. This review summarizes several kinds of these emerging PTMs, including the description of proteins to be modified, the function of PTMs, the enzymes that control them, the technologies to study them and the potential therapy targets. According to different types of modified functional groups, the newly reported PTMs with TME remodeling function are divided into the following types: protein acylation modification, lipid-related protein modification, metabolite-related protein modification, and ubiquitin-like small-molecule protein modification. This review generally described the effect of some PTMs on tumorigenesis and cancer development by affecting the regulatory mechanism of growth and metabolism, immune response, and mechanical stress in tumor microenvironment. Direct targeting tumor microenvironment is the most promising method of tumor therapy, which has great basic and clinical research potential. In-depth analysis of PTM of different target proteins in TME will bring new breakthroughs for effectively relieving immunosuppression and promoting the clinical efficacy and wide application of tumor immunotherapy.

## Protein-acylation modification

Acyl CoA compounds are important metabolic intermediates in cells and precursors of many biological macromolecules. With the development of research technology and the deepening of research level, the role of protein acylation modification in the regulation of TME has been gradually explored.

### Acetylation

Protein acetylation refers to the transfer of the acetyl group from acetyl coenzyme to lysine under the catalysis of acetyltransferase, which was first reported to occur in the lysine-rich region of the N-terminal of histone.^24^ Subsequently, the modification-related target proteins, acetyltransferases, deacetylases, and their roles in cell biological regulation were gradually reported. From the perspective of epigenetics, there is a clear causal relationship between histone acetylation and gene transcriptional regulation, including acetylation in nonhistone proteins involved in transcriptional regulation.^25^ Numerous studies have shown that the protein acetylation at lysine residue is mediated by lysine acetyltransferases (KATs) and deacetylation is mediated by deacetylases (KDACs). Thousands of human histone or nonhistone proteins are characterized to be acetylated through high-resolution MS-based proteomics, in combination with the enrichment of acetylated peptides.^26^ A large number of studies have gone deeper in the characterization of the protein lysine acetylation.^27,28^ The intermediate products of cell metabolism dominated by acetyl coenzyme A directly regulate protein acetylation.^29^ Non-acetyl metabolites can also regulate deacetylases, such as the deacetylation of dinucleotide involved in cell electron transport chain catalyzed by NAD^+^-assisted sirtuin.^30^ The details will be described in the following text.

### Propionylation

Propionylation is another acetylation-like modification identified by MS, which has been well demonstrated in both prokaryotes and eukaryotes. The bromodomain and plant homeodomain (PHD) finger containing protein 1 (BRPF1) belongs to monocytic leukemic zinc finger (MOZ) histone acetyltransferase (HAT) and recognizes histone acetyl lysine marks to affect gene transcription.^31^ Lysine acetyltransferase 6A (KAT6A) has essential roles in histone acetylation to regulate chromatin organization and function.^32^ Recently, BRPF1-KAT6 complexes were identified as propionyltransferases both in vitro and in vivo. As the dual targets of both acetylation and propionylation, histone H3K23 is associated with distinct chromatin impact in response to acetyl-CoA and propionyl-CoA, respectively.^33^ Acetyltransferases p300 and CBP were found to carry out auto-propionylation. Histone deacetylase sirtuin 1 (SIRT1) is responsible for depropionylation of p53 and p300.^34^ They may regulate cellular metabolism since the substrate propionyl-CoA is derived from odd-chain fatty acid and amino acid catabolism.^35^

### Butyrylation and isobutyrylation

**Fig. 1:**
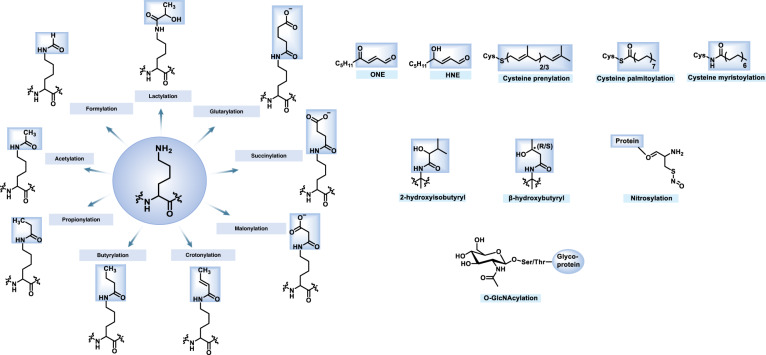
Chemical structures of histone/nonhistone PTMs in this review

Besides acetyl-CoA and propionyl-CoA, butyryl-CoA also belongs to high-energy molecules to be the modification substrates in lysine side chain formed during the oxidation of fatty acids (Fig. 1). Lysine butyrylation (Kbu) was first discovered by Zhao et al. with synthetic peptides and in vitro enzymatic reactions as a novel lysine modification dependent on n-butyryl-CoA and catalyzed by acetyltransferases.^35^ Studies demonstrated that p53 can be propionylated and butyrylated in vitro, catalyzed by p300 and CBP.^34^ This modification can be reversed by the HDAC inhibitor suberoylanilide hydroxamic acid (SAHA) with a growth inhibition of neuroblastoma.^36^ Isobutyryl-CoA is another intermediate derived from branched-chain fatty acid oxidation, leading to lysine isobutyrylation by isobutyryltransferase p300 and HAT1.^37,38^ Lysine 2-hydroxyisobutyrylation (Khib) in histone H4K8 is abundant and associated with active transcription in male germ cells, opening a new window to study the potential functions of this new modification.^39^

### Crotonylation

Lysine crotonylation (Kcr) is first identified by Zhao et al. among all core histone proteins including H2A, H2B, H3, H4, and linker histone H1. This modification shows a distinct genomic distribution pattern from acetylation with its specific label property of TSS of active genes.^40^ The transcriptional coactivator and HAT p300 is reported to have histone crotonyltransferase (HCT) activity. P300-catalyzed histone crotonylation promotes enhanced transcription effect than p300-catalyzed acetylation. In this study, the nucleic/cytosolic acetyl-CoA synthetase enzyme (ACSS2) was implicated to produce intermediate product crotonyl-CoA from the short-chain fatty acid (SCFA) in mammalian cells.^41^ ACSS2 is overexpressed with the function in acetate uptake and lipid conversion in human cancer. The inhibition of tumor growth and failure of glioma self-renewal were found in ACSS2 deficient mice.^42,43^ A large number of nonhistone proteins can also be modified by crotonylation in the regulation of subcellular location, cellular composition and function, signal pathways, and biological processes.^44^ After crotonic acid treatment, serine 46 residue in p53 was identified to be crotonylated other than other protein family members p63 and p73. This directly led to reduced p53 level and consequently abnormality in glycolysis pathway and mitochondrial activity.^45^ Lysine crotonylation plays an inhibitory role in the development and metastasis of hepatocellular carcinoma.^46^ Sirtuin family members SIRT1, SIRT2, and SIRT3 are responsible for the removal of histone H3K4 crotonylation in vitro. In addition, SIRT3 selectively targets histone crotonylation and functions as “eraser” to regulate histone crotonylation level and gene expression in living cells.^47^ Crotonylation is identified preferentially by YEATS (Yaf9, ENL, AF9, Taf14, Sas5) family proteins.^48^ The co-crystal structure of AF9 YEATS domain in complex with H3K9 acetylation and crotonylation defined a higher affinity of YEATS for crotonyl-lysine, linking histone crotonylation to active transcription.^49^

### β-hydroxybutyrylation and β-hydroxyisobutyrylation

Another kind of modification of histone lysine derived from endogenous short-chain fatty acid metabolism is β-hydroxybutyrylation/β-hydroxyisobutyrylation, also known as 2-hydroxybutyrylation/2-hydroxyisobutyrylation, exists in a variety of bio-fluids in humans. Lysine acetyltransferases p300 is responsible to catalyze both acetylation and 2-hydroxyisobutyration reactions on histones with different activity. A global Stable Isotope Labeling by Amino Acids in Cell Culture (SILAC)-based assay and MS analysis were used to define distinct target protein sequence between p300-mediated acetylation and 2-hydroxyisobutyration. Interesting, most of the β-hydroxyisobutyrylated proteins are glycolytic enzymes, revealing this p300-mediated modification is essential for cellular glucose metabolism regulation.^50^ Both in vivo and in vitro studies reflect the regulation role of human MYST family acetyltransferase Tip60 in β-hydroxyisobutyrylation at sites H4K8, H4K12, and H4K16. In addition, the removing of β-hydroxyisobutyrylation is mediated by histone deacetylases 2 (HDAC2) and HDAC3.^51^ However, the Class III sirtuins family member Sirt3 appears to have site-specific activity of β–hydroxybutyrylation in H3K4, K9, K18, K23, K27, and H4K16, which is distinct from the Zn-dependent HDACs.^52,53^

### Malonylation

The property of electron-rich and nucleophilic nature in lysine side chain makes it suitable to undergo covalent post-translational modification, especially bulkier groups carrying negatively charged carboxyl moieties. Lysine malonylation (Kmal) refers to the incorporation of a malonyl group at the ε-amine and this modification was first validated by Zhao et al. in HeLa cells and *Escherichia coli* (*E. coli*).^54,55^ Even the anti-malonylysine antibody is reasonable to detect malonylated proteins, this technology is restricted for tracing the dynamic of malonylation. In another study, an alkyne-functionalized chemical probe was used to profile more than 300 malonylated substrates.^56^ Abnormal metabolites are associated with many diseases. For example, elevated malonyl-CoA level was found in type 2 diabetic patients as well as type 2 diabetes model *db/db* mice.^57,58^ Sirt5 was responsible for demalonylation. Increased protein malonylation was observed in mice lacking Sirt5, which was involved in multiple metabolic networks like glycolysis, gluconeogenesis, urea cycle, and fatty acid β-oxidation.^59^

### Succinylation

Protein succinylation reaction was first identified in bacterial metabolism.^60^ Thousand of succinylation sites were mapped in diverse organisms including bacteria (*E. coli*), yeast (*S. cerevisiae*), human (HeLa) cells, and mouse liver tissue through high-resolution quantitative MS and antibody-based affinity enrichment followed by strong cation exchange (SCX) chromatography.^61^ Histone succinylation was validated by MS/MS of synthetic peptides, HPLC co-elution, and in vivo isotopic labeling with the enzyme-mediated cofactor succinyl-CoA. This PTM is high conserved from *Saccharomyces cerevisiae*, *Drosophila* S2, mouse embryonic fibroblast, and human cells.^55^ Lysine succinylation may cause the change of charges from 0 to −2, the same effect caused by protein phosphorylation at serine. It is expected that succinylation could have important cellular functions.^62^ A recent study demonstrated SIRT5 is the major deacylases for lysine desuccinylation.^63^

### Glutarylation

The type of lysine glutarylation (K_glu_) was validated as an evolutionarily conserved PTM, in which SIRT5 showed deglutarylation activity both in vitro and in vivo.^63,64^ Carbamoyl phosphate synthetase 1 (CPS1), the key enzyme important for ammonia-detoxifying urea cycle, was identified to be a glutarylated substrate with inhibit enzyme activity after glutarylation.^63,65^ Other than MS, the novel bioinformatics tools, the biased support vector machine algorithm, and machine-learning scheme are discovered to predict the potential glutarylation sites and identify physiochemical and sequence-based features.^66–70^ Glutarylation in mitochondrial proteins suppresses glutamate dehydrogenase (GDH) activity and protein interactions.^71^

## Lipid-related protein modification

Lipids are fundamental structural components of cellular membranes, acting as barriers to separate oneself from the external environment and to divide cells into different functional areas. The interaction of some proteins with specific lipid molecules and the covalent modification of lipid molecules of proteins are two mechanisms of lipid-dependent cell signal transduction in cell. In humans, some proteins undergo more complicate modification such as the addition of lipids to increase plasma membrane docking or regulate protein complex formation. Lipidation modification includes C-terminal glycosylphosphatidylinositol (GPI) anchor, N-terminal myristoylation, S-palmitoylation, and S-prenylation. In this section, we summarize three lipid modification types including myristoylation, palmitoylation, and prenylation.

### Myristoylation

Protein myristoylation refers to the attachment of a 14-carbon fatty acyl group myristic acid to target substrates via an amide bond, which is irreversible and mediated by the eukaryotic enzyme myristoyl-CoA: protein N-myristoyltransferase (NMT).^72^ Two NMTs are found in human cells (NMT1 and NMT2) according to their protein size.^73^ This modification exits widely in viral, yeast, plant, and eukaryotic proteins to mediate signaling cascaded.^72,74,75^ Many enzymes in metabolic pathways undergo this modification, supporting its importance and specific regulatory roles. As myristic acid is a hydrophobic group, the myristoylated proteins are inserted into lipid rafts in the plasma membrane, including endoplasmic reticulum (ER), Golgi apparatus, mitochondria, and nuclear to regulate diverse cellular functions.^76^ A number of myristoylation sites related to tumors have been identified (Table 1).

**Table 1:** Identified protein myristoylation sites related to tumors

Target	Function in cancer	Ref.
LAMTOR1	Inhibits lysosomal degradation.	^588^
FMNL1/3	Controls cytoskeleton remodeling and cellular morphological changes.	^202,465^
SFKs	Promotes membrane attachment and Src-family kinase activity.	^256^
TRAM	Enhances the membrane attachment for LPS and TRL7 signaling.	^432^
Fyn	Promotes subcellular location in immunological synapses and interaction with TCRζ chain.	^459,460^
c-Src	Regulates the activity of FAK in cell adhesion and extracellular matrix.	^72,461^
Lck	Promotes TCR signal cascade.	^448^
eNOS	Regulates enzyme activity and signal transduction.	^470^
Fus1	Facilitates protein docking to the mitochondrial membrane.	^505^
BID	Improves its insertion into mitochondria membrane to enhance apoptosis.	^506^
PAK2	Promotes protein location in membrane ruffles and internal membranes.	^507^
SAMM50, TOMM40, MIC19, and MIC25	Promotes mitochondrial localization and membrane binding.	^589^
AMPK	Helps the removal of damaged mitochondria by autophagy.	^328^
AMPK	Enhances the β oxidation of fatty acids.	^590^
gelsolin	Regulate nuclear fragmentation or cytoskeletal remodeling during apoptosis.	^301^
DIC2		
Bap31		
MACF		
YTHDF2		

**Fig. 2:**
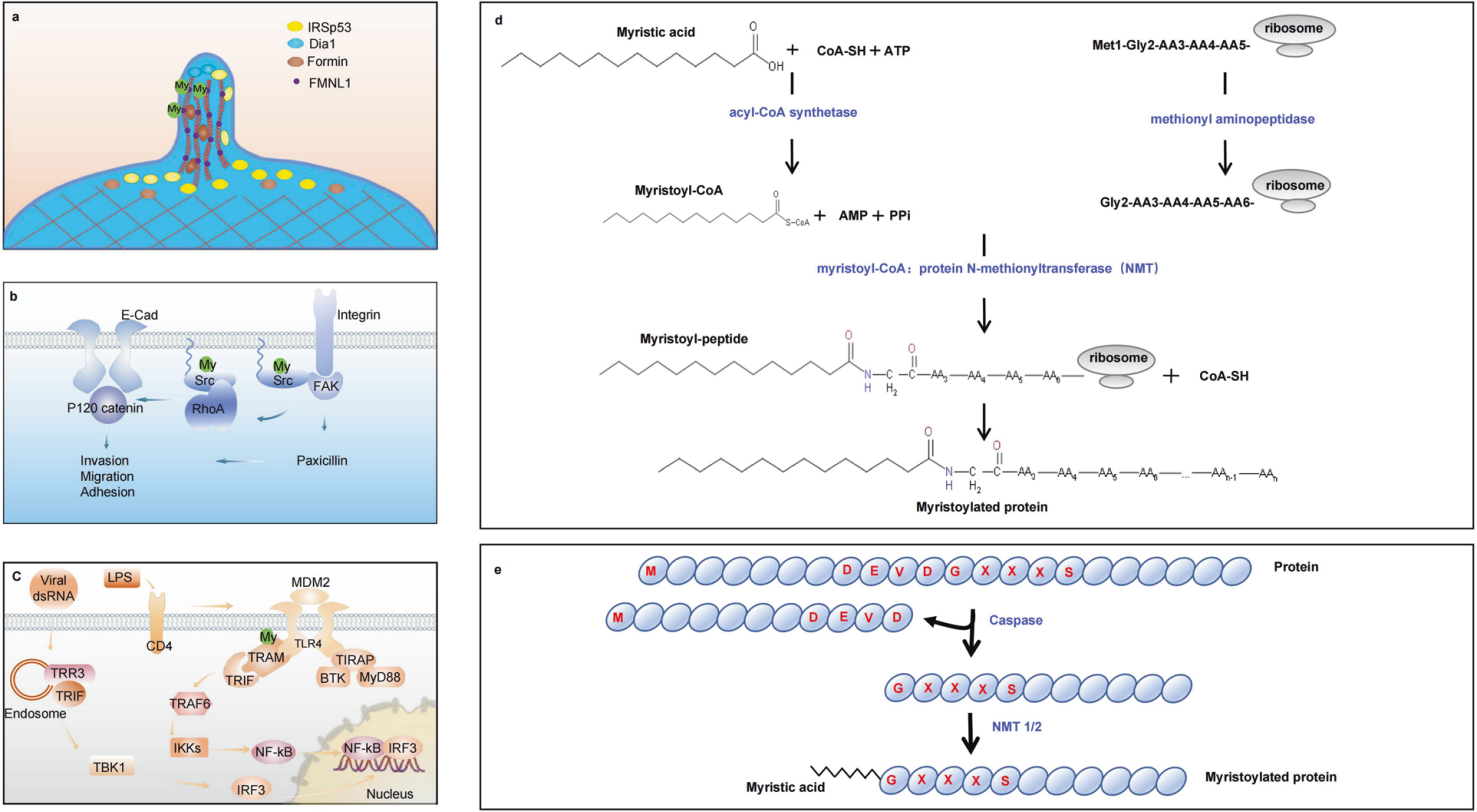
Schematic representation of protein N-myristoylation and function. **a** The myristoylation of formin family protein FMNL1 leads to membrane association, membrane trafficking and bleb formation. **b** The N-terminal myristoylation is essential for the membrane attachment and kinase activity of Src. **c** The myristoylated TRAM tethers it to the membrane, serving as a prerequisite for LPS signaling. **d** Co-translational N-myristoylation. The initiator methionine is removed by methionine aminopeptidase 2 and myristic acid is transferred to the N-terminal glycine residue by NMT. **e** Post-translational N-myristoylation. Some proteins are cleaved by a protease to facilitate a myristic acid is covalently attached to glycine residue by NMT

In addition, N-glycine myristoylation-mediated protein–protein and protein-membrane interactions change the subcellular localization of targeted proteins across various cellular processes, suggesting a promising therapy target^77^ (Fig. 2). A protease from macrophage extracts was reported to cleave myristoylated alanine-rich C kinase substrate (MARCKS), indicating myristoylation can be a substrate for protease.^78^ The demyristoylation of MARCKS was also observed in cytoplasmic fraction of synaptosomes from the bovine brain with the property of ATP and coenzyme A (CoA) dependence.^79–81^ Moreover, bio-orthogonal probes and chemical tools, especially the non-radioactive-labeled fatty acids like ω-azido or ω-alkynyl myristate, are effective and easily applicable to identify N-myristoylated proteins.^82–85^ Recent studies have found the phase separation-related myristoylationon of tumor growth, expanding the mechanism research and treatment direction of lipid-associated protein modification. The oncogenic activity of lysine methyltransferase EZH2 relays on methylation and further phosphorylation of its substrate STAT3.^86,87^ The hydrophobic effect of EZH2 myristoylation promotes droplets formation to separate from the surrounding solution, which is conducive to the interaction with STAT3 and downstream growth regulation.^88^ N-Myristoylation Predictive Tools are developed based on the PROSITE motif in substrate proteins^89^ (http://mendel.imp.univie.ac.at/myristate/), the difference between myristoylated and non-myristoylated proteins^90^ (http://www.expasy.org/tools/myristoylator/myristoylator.html) and pattern scanning^91^ (http://www.isv.cnrs-gif.fr/terminator3/index.html).

### Palmitoylation

Protein palmitoylation, often occurs on cysteine and also known as S-palmitoylation, is the addition of a 16-carbon palmitoyl group to target proteins through thioester bonds. This reaction is reversibly catalyzed by palmitoyl-CoA palmitoyltransferases or non-enzymatic acylation and the converse is depalmitoylase or non-enzymatic hydrolysis.^77^ The conserved family of protein acyltransferases (PATs) are responsible for protein palmitoylation. They are firstly discovered encoded by the ERF2 and ERF4 genes targeting Ras GTPase.^92^ In mammalian cells, 23 members of the Golgi-apparatus-specific protein family with the aspartic acid-histidine-histidine-cysteine (DHHC) zinc finger domain are identified to have PAT activity.^93,94^ In addition, DHHCs generally show substrate specificity and more than one DHHC modify a palmitoylated substrate.^94–97^ Most mutations of DHHC enzymes are associated with neurodegenerative diseases like Huntington’s, Alzheimer’s, X-linked mental retardation, and schizophrenia, as well as other developmental defects and cancer.^77^ For example, the depletion of palmitoyltransferase DHHC5 inhibits NSCLC cell growth, colony formation, and cell invasion, indicating the potential of oncogene function of DHHC5.^98^ Studies provided a dynamic loop between palmitoylation and depalmitoylation, which has been emerging as an important regulation mechanism for cellular homeostasis. Two acyl-protein thioesterase-1 APT1 (also called LYPLA1) and APT2 (also called LYPLA2) catalyze depalmitoylation of palmitoylated proteins anchored in the membrane.^99^ The radioactive-isotope-labeled palmitic acid and the following immunoprecipitation are effective methods to detect protein palmitoylation.^100^ Later, the higher sensitive bio-orthogonal probes, which contain a terminal azido or alkynyl group, are developed to obtain precise detection.^101^ Then a three in vitro steps of acyl-biotin exchange (ABE) assay were developed to detect palmitoylation under any conditions.^102^

### Prenylation (farnesylation + geranylgeranylation)

Protein prenylation refers to the addition of different isoprene unit to C-terminal cysteine residues. The attachment of farnesyl diphosphate (FPP) forms farnesylation and the attachment of geranylgeranyl diphosphate (GGPP) forms geranylgeranylation. These two different sizes of polyisoprenoid groups were identified in Chinese hamster ovary (CHO) cells.^103^ Furthermore, the geranylgeranyl-modified proteins were discovered in HeLa cells through the analysis of radioactive mevalonic acid-labeled fragments.^104^ About 2% of total cellular proteins are prenylated in eukaryotic cells with the majority are geranylgeranylated proteins.^105^ It was reported that protein geranylgeranylation is required for cell-cycle transition from G1 to S phase, but not farnesylation.^106^

Farnesyltransferase (FT), geranylgeranyltransferase (GGT-1), and Rab Geranylgeranyl transferase (RGGT or GGT-2) are the major protein prenyltransferase.^77^ The first FT was characterized from rat brain cytosol with the function of transferring 15-carbon farnesyl group farnesyl moiety from farnesyl pyrophosphate to p21RAS cysteine residue.^107^ In addition, GGT-1 mainly transferred a 20-carbon geranylgeranyl group to substrates and has different preference with FT.^108^ GGT-2 functions in the linkage of 20-carbon geranylgeranyl groups to cysteine residues in Rab proteins.^109^ Some website tools are available for protein prenylation prediction such as Prediction Suite (http://mendel.imp.ac.at/PrePS/). Even the thioether bond is stable between farnesyl or geranylgeranyl groups and cysteine, a kind of prenylcysteine lyase located in lysosomal was identified to cleave prenylated proteins.^110,111^

## Metabolism-associated protein modification

### Lactylation

According to the restricted oxygen supply in the tumor microenvironment, hypoxia becomes a key hallmark in solid tumors and also promotes tumor progression. Tumor cells have increased glucose uptake and consistent lactate production even in the presence of oxygen (Warburg effect).^112^ This transition from oxidative phosphorylation (OXPHOS) to an altered glycolysis is regulated by a large number of oncogenes and tumor suppressor genes. Lactate in tumor cells comes from two sources: the pyruvate conversion in the last reaction of glycolytic pathway, and the conversion of phosphoenolpyruvate (PEP) by a tumor-specific isoform of pyruvate kinase (PK) M2.^113^ Recent data suggest that the lactate may be involved in many biological processes, including epigenetic regulation, intrinsic inflammatory mediation, immunologic escape, tumor cell motility and migration, O_2_ independent angiogenesis, radioresistance or chemoresistance, acidic or hypoxia tumor microenvironment, and extracellular matrix remodeling.^113^ The detailed mechanisms of lactate regulation in tumor development and immune response are summarized previously^113–119^ and we discuss the newly identified protein lactylation in this review. Lysine lactylation was identified by Zhao et al. after the stimulation of lactate with high-performance liquid chromatography (HPLC)-tandem mass spectrometric (MS/MS) analysis.^120^ Lactic acid from extracellular glucose or hypoxia induced glycolysis promotes histone lactylation and directly regulates gene transcription.^120^ In addition to the regulation function of lactic acid as a metabolite intra- and extracellular signal molecular on innate and adaptive immunity in the microenvironment,^121^ protein lactylation plays a role in gene transcription and immune adaptation. The details will be described later.

### O-GlcNAcylation

The covalent attachment of glycans to proteins is called protein glycosylation, which is a common contribution to structure and modification diversity.^122^ Protein GlcNAcylation refers to the addition of single N-acetylglucosamine residue O-linked to the hydroxyl group of serine and threonine residues of multiple nuclear and cytosolic proteins, including transcription factors, tumor suppressors, nuclear pore proteins, kinases, and cytoskeleton proteins. These modified proteins are involved in signal pathway, metabolism, transcriptional, DNA replication, protein trafficking, and other cellular processes.^123,124^ Multistep radioactivity-based approach and quadrupole time-of-flight (Q-TOF) MS are used to identify the modification sites.^125^ Since it was first identified on lymphocyte proteins, it was paid more and more attention to support its dynamic regulatory function.^123,126^ A UDP-N-acetylglucosamine: peptide N-acetylglucosaminyltransferase (O-GlcNAc transferase, OGT) and a neutral and cytoplasmic N-acetyl-β-d-glucosaminidase (O-GlcNAcase, OGA) are responsible for the addition and removal of O-GlcNAc to target proteins, respectively.^127,128^

### Nitrosylation

Nitric oxide (NO) is an important biological second messenger in the regulation of cardiovascular pharmacology, cell migration, inflammatory response, and neurobiology.^129^ This highly reactive molecule is synthesized by NO synthase (NOS) with l-arginine and molecular oxygen as substrates.^130^ Protein S-nitrosylation refers to the attachment of an NO group to a reactive cysteine residue, leading to the formation of an S-nitrosoprotein (SNO-protein).^131^ Dysregulated SNOs are associated with diseases through the influence of nitrosothiol formation, processing, and degradation.^132,133^ It has become clear that protein S-nitrosylation and denitrosylation play an important role in protein–protein interaction, subcellular localization, and degradation, thus regulating a wide range of cellular processes, including transcription, ion-channel activity, metabolism, cellular redox maintenance, apoptosis, and other kinds of PTM.^134^

## Ubiquitin-like small-molecular protein modification

There exist one kind PTM refers to the conjugation of small peptides by a reversible cycle on the substrates such as the well-known small ubiquitin and ubiquitin-related modifier family in eukaryotic cells. Protein modification by this small-molecule conjugation influence all parts of cellular processes such as mitochondrial function, chromosome remodeling, DNA repair, transcriptional regulation, nuclear transport of intranuclear proteins, heterochromatin construction, plasma membrane association, cytoskeleton dynamics, and protein aggregation.^135,136^ In addition, humans have a set of ubiquitin-like proteins (UBLs) that covalently modify target proteins.^137,138^ These UBLs, including ubiquitin-fold modifier 1 (UFM1), Neural precursor cell expressed, developmentally downregulated 8 (Nedd8), small ubiquitin-like modifier 1 (SUMO-1), SUMO-2, SUMO-3, Fau gene-encoded ubiquitin-like protein (FUBI), homologous to ubiquitin 1 (HUB1), interferon-stimulated gene 15 (ISG15), The human leukocyte antigen F-associated transcript 10 (FAT10), ubiquitin-related modifier 1 (URM1), autophagy-related 8 (ATG8), and ATG12, are found in prokaryotes, archaea, yeast, plants, and mammals.^139^ Moreover, the Ub domain proteins (UDPs) also have a role linking the ubiquitination with the functions of the proteasome. These include the proteasome-associated deubiquitinating enzyme UBP6, Rad23, the DSK2-relative PLIC2/Chap1, BAT3/Chap2, BAG-1, elongin B, and parkin.^140^ A multistep enzymatic cascade, made up of ATP-dependent activation enzyme E1, binding enzyme E2 and ligase E3, are required in the covalent addition of ubiquitin-like small-molecular protein via the isopeptide bonds between C-terminal diglycine motifs and ε-amino groups corresponding substrates.^141^ Several E1, dozens of E2, and hundreds of E3 are encoded in the human genome.^142–144^ As a reversible process, the deubiquitinases (DUBs; also known as deubiquitylating or deubiquitinating enzymes) can remove the UBLs modification from target proteins and break the ubiquitin chains.^145,146^ These DUBs are divided into six families: ubiquitin-specific proteases (USPs), herpesvirus tegument USPs (htUSPs), ubiquitin C-terminal hydrolases (UCHs), ovarian tumor proteases (OTUs), the family of Josephins and the JAB1/MPN/MOV34 proteases (JAMMs).^139^ Many of these enzymes in UBL modification are identified by labeling dependent and independent quantitative proteomics, such as the reported stable isotope labeling by amino acids(SILAC) in cell culture through metabolic incorporation,^147,148^ isobaric tags for relative and absolute quantification (iTRAQ),^149^ and the absolute quantification (AQUA) strategy.^150,151^

### UFMylation

UFM1 is a 9.1-kDa protein with a similar structure to ubiquitin.^152^ It conjugates into target proteins through E1, E2, and E3 enzymes. These refer to the UFM1-activating enzyme (ubiquitin-like modifier-activating enzyme 5; UBA5), the UFM1-conjugating enzyme 1 (UFC1), and the UFM1-specific ligase 1 (UFL1), respectively.^153^ Protein UFMylation is reversible and conserved among nearly all the eukaryotic organisms, except for yeast and is involved in the homeostasis of the ER stress response, cell differentiation, and NF-κB binding protein-related cell-cycle control.^154^ UFM1 is cleaved from target proteins by the UFM1-specific proteases (UfSPs).^155^ Recent studies have demonstrated that UFMylation plays a role in the regulation of ER stress,^156^ vesicle trafficking,^157^ cell-autonomous erythroid differentiation,^158^ hematopoiesis and development,^159^ β-oxidation of fatty acids,^160^ and G-protein-coupled receptor (GPCR) biogenesis.^161^

### Neddylation

With the highest amino acid similarity to ubiquitin (identical 58%) of NEDD8 (neural precursor cell-expressed developmentally downregulated 8), protein neddylation is widely studied in cancer. This modification includes the E1 NEDD8-activating enzyme (NAE, composed of NAE1 (APP-BP1) and Uba3 heterodimer), the E2 NEDD8-conjugating enzyme Ubc12 (also known as Ube2m) and Ube2f, and the RING finger domain-containing E3s.^162^ The Cullin family (Cul1–5, Cul7), PARC, p53/p73/BCA3/VHL, MDM2, and EGFR are substrates of neddylation and functions in the regulation of a set of cellular processes, including cell-cycle inhibition, HIFα degradation, cytokine modulation, oxidative stress-response pathway, DNA replication and nucleotide excision repair, p53 family location and antiapoptotic function of oncogenes.^162^ In addition to Cullins, the cytoskeleton was another target of Nedd8 conjugation to facilitate the transition from meiosis to mitosis in *Caenorhabditis elegans.*^163^ Deneddylation is mediated by the Jab1/MPN domain metalloenzyme (JAMM) motif in zinc metalloprotease COP9 signalosome (CSN) and cysteine protease NEDP1 through conformational changes to promote rapid remodeling of cellular neddylation and deneddylation cycle.^164,165^ The HEAT repeats protein CAND1 (Cullin-associated and neddylation-dissociate 1) inhibits neddylation through forming a ternary complex with CUL1 and ROC1 and disrupting the linkage between NUB1 (NEDD8 ultimate buster 1) and 26S proteasome and the latter proteasomal-degradation pathway.^166–169^ Other deubiquitinlase showed deneddylases activity, including ubiquitin-specific peptidase 21 (USP21), deubiquitylating enzyme Ataxin-3 (ATX3), *P. falciparum* deubiquitinating enzyme PfUCH54, ubiquitin C-terminal hydrolase L1 (UCH-L1), and UCH-L3.^162,170,171^

## The biological effect of PTMs on tumor cells in TME

### Chromatin organization and gene transcription

Histones are building blocks of nucleosome, the fundamental part of chromatin. The unstructured N-terminal tail offers a massive opportunity for a large number of PTM. Histone PTM influence various biological processes through two major mechanisms. First, different PTM regulate the interaction between nucleosomes with other nonhistone proteins, leading changes in downstream functions like transcription, replication, and repair. Second, PTM can affect the interaction between nucleosomes with adjacent DNA through the changed properties like net charge, hydrogen bonding, size, or hydrophobicity, thus altering higher-order chromatin structure and DNA-based biological function.^172^

According to the chemical structures and properties, propionylation (three-carbon molecule: C3) and butyrylation (C4) are the most similar PTMs to acetylation (C2). The site-specific antibodies are effective to characterize propionylation and butyrylation. Histone PTMs at different sites are linked with activation or suppression of transcription through chromatin organization. Lys 23 of histone H3 (H3K23) is detected to be propionylated in the leukemia cell line catalyzed by the histone acetyltransferase p300, promoting an active chromatin structure to regulate transcription even with different dynamics of acetylation.^173^ Recently, BRPF1 (bromodomain- and PHD finger-containing protein 1) were reported to activate lysine acetyltransferase 6A (KAT6A) and KAT6B, leading to the propionylation of histone H3K23 in vitro and in vivo.^33^ Lys 14 of histone H3 (H3K14) is considered to be a hallmark of transcriptional activation by the directed recruitment of basal transcription factor TFIID with the demethylation of transcription co-repressor ZMYND8.^174,175^ A MS analysis identified propionylation and butyrylation in histone H3K14 with a strong linkage with transcriptional activation. Both p300 and the GNAT family HATs, GCN5 and PCAF have acyltransferase activity both in vitro and in vivo.^176^ The butyrylation activity of p300 was also confirmed in H4K5 and H4K8 and histone butyrylation was observed to directly stimulate gene transcription.^177^ The isobutyrylation (2-methylpropionylation) is an isomeric structure of butyrylation, taking intermediate product f isobutyryl-CoA generated from the valine metabolism pathway. KAT enzymes, particularly the acetyltransferase p300 and HAT1, are identified to catalyze both lysine propionylation and lysine isobutyrylation to mediate histone H4 isobutyrylation.^38^ In addition to CBP and p300, another histone acetyltransferases MOF was also showed HCT activity linking with TGFβ-induced transcriptional activation.^178^ Recently, class I HDACs were demonstrated to possess histone decrotonylation activity in cells. According to the transcription level of six housekeeping genes by qRT-PCR, this histone decrotonylation could repress global transcriptional repression through the disruption of promoter recruitment of crotonylation reader proteins.^179^ Histone lysine β-hydroxyisobutyrylation is identified at multiple sites in response to β-hydroxybutyrate level with cofactor b-hydroxybutyryl-CoA. Starvation-induced Kbhb at H3K9 may alter chromatin structure to form an active transcription pattern.^180^ In another study on mice and cells, histone3-lysine9-β-hydroxybutyrylation (H3K9bhb) was induced by fatty acid metabolism-associated β-hydroxybutyrate to promote the expression of growth factor BDNF, thereby regulation depression.^181^

Histones are identified to be glutarylated in human Hela cells, especially the histone H4 at site Lys91 (H4K91glu). The evolutionarily conserved modification of H4K91glu may inhibit the assembly of H2A-H2B dimers to the H3-H4 tetramer to form histone octamer. The release of H2A-H2B from nucleosome was also observed after glutarylation with a fluorescence resonance energy transfer (FRET) assay.^182^ Moreover, the mutation of H4K91E, which mimic H4K91glu, resulted in significantly delayed S and G2/M cell cycle, increased sensitivity of DNA damage molecules methyl methanesulfonate (MMS), hydroxyurea (HU), and UV radiation, as well as a less compact chromatin signature.^182^ Through the screen of all HDACs (HDAC 1–11, sirtuin 1–7) for lysine deglutarylation activity, Sirt5 was found to be a deglutarylase on mitochondrial proteins.^63^ Even Sirt5 had no function in H4K91 glutarylation, SIRT7 examined the histone deglutarylase activity both in vitro and in cells upon chromatin condensation.^182^ KAT2A, also known as GCN5, belongs to HAT family. 2-Oxoglutarate dehydrogenase (OGDH) is the E1 subunit of α-ketoglutarate dehydrogenase (α-KGDH) complex, that catalyzes α-ketoglutarate (α-KG) conversion into succinyl-CoA.^183^ After the interaction between KAT2A and OGDH, the binding affinity of succinyl-CoA was observed, leading to a significant histone H3 succinylation.^184^ In addition, this interaction also promoted H3K79succ and H4K91glu with the co-factors succinyl-CoA and glutaryl-CoA, respectively. The various function of KAT2A in histone acetyltransferase, succinyltransferase, or glutaryltransferase have highly suggested the tight link between cellular metabolism and epigenetic regulation.^182^

Histone O-GlcNAcylation regulates chromatin configuration to influence gene expression and DNA repair. Histones and other proteins responsible for DNA assembly are found to be O-GlcNAcylated in vivo, suggesting the role of O-GlcNAcylation as part of the histone code.^185^ GlcNAcylation of histone H3K4 methyltransferase 5 (MLL5) at Thr440 promote its methylation state, leading to a transcriptionally active chromatin. Histone H2B was reported to serve as an OGT substrate in response to serum glucose stimulation in S112 site. This histone GlcNAcylation may facilitate transcription considering it was located near the transcription start site of many genes.^186^ Through co-immunoprecipitation and MS analysis, ten-eleven translocation (TET) enzyme TET2 was identified directly interacting with OGT at transcription starting sites (TSS). This interaction not only increases histone O-GlcNAcylation but also regulate gene transcription.^187^ Moreover, the induction of DNA double-strand breaks (DSBs) increased histone H2B O-GlcNAcylation both in homologous recombination (HR) and non-homologous end-joining (NHEJ) pathway. Functionally, O-GlcNAcylated H2B bind and recruited DNA damage repair protein Nijmegen breakage syndrome 1 (NBS1) and regulated NBS1 foci formation.^188^

The transcriptional activity of p53 and its homolog TAp73 is negatively regulated by neddylation. The neddylated MDM2 RING finger E3-ubiquitin ligase promotes p53 neddylation to abrogate its transcription function, without a significant influence on MDM2-mediated p53 degradation.^189^ The p53 family member p73 contains various isoforms and the full length (TAp73) can induce cell-cycle arrest and apoptosis.^190^ TAp73 was covalently neddylated in an MDM2-dependent manner and deneddylated by NEDP1. TAp73 neddylation promoted its location in the cytoplasm and attenuated transactivation function.^191^ In addition to MDM2, the E3-ubiquitin ligase F-box only protein 11 (FBXO11), is also responsible for p53 neddylation and suppression of p53 transcriptional activity.^192^ Ribosomal proteins (RPs) have been identified to interact with MDM2 and negatively regulate p53 under stress signals. Recently, RPL11 are identified to be neddylated in a MDM2-depended manner to avoid destabilization with proteomic approach.^193^ Moreover, the interaction with MDM2 and the related neddylation of RPL11 triggered a rapid and transient recruitment at promoter sites of p53-regulated genes under actinomycin D (ActD) induced nucleolar stress. This reversed MDM2-mediated p53 transcriptional repression.^194^ The E2F transcription factor family regulates cell proliferation, cell-cycle transition, differentiation, and apoptosis in response to DNA damage as either activators or repressors.^195^ Protein interaction and various PTMs regulation are important for E2F1 transcription activity.^196^ E2F1 was methylated by Set9 at Lys185 to prevent its accumulation at DNA damage sites and downstream gene p73 activation. LSD1 functioned in demethylation of E2F1 to rescue its apoptotic function.^197^ The phosphorylation of E2F1 was mediated by checkpoint kinase 2 (Chk2) after DNA damage, leading to protein stabilization and proapoptotic transcriptional activation.^198^ The acetyltransferase p300 is responsible for acetylation and ubiquitination of E2F1.^199^ E2F1 was later identified to be a new substrate for neddylation, with a negatively regulated transcriptional activity and DNA binding.^200^

### Protein–protein interaction

More and more studies have demonstrated that protein lipidation is essential for protein interactions. The functional role of myristoylation has been reported since many N-myristoylated proteins were key players in various cellular processes. According to the crystal structure of Ca^2+^-CaM binding with myristoylated peptide derived from N-terminal domain of CAP-23/NAP-22, the function of myristoylation in protein–protein interaction was first defined.^201^ The inhibition of myristoylation of Formin family proteins FMNL1 and FMNL3 led to cytoskeleton remodeling and later cellular morphological changes.^202^ Human SOS (hSOS1) is a Ras guanine-nucleotide exchange factor and this activity is highly dependent on protein prenylation.^203^ The guanine-nucleotide exchange activity appeared on prenylated K-Ras(4B) but not on unprocessed K-Ras(4B) under the stimulation of growth factors.^204^ The geranylgeranylation of RhoA regulates its interaction with GDP-dissociation inhibitor (GDI) and GDP-dissociation stimulator (GDS).^205^ The prenylated RhoA also showed a significant interaction with IQ-motif-containing GTPase-activating protein IQGAP1 to regulate breast cancer cell proliferation and migration.^206^

Recent work revealed protein–protein interaction may be inhibited or activated by O-GlcNAcylation. O-GlcNAcylation modification of transcription factor Sp1 regulated transcription by decreasing its interaction with TATA-binding-protein-associated factor (TAF110) both in vivo and in vitro.^207,208^ In addition, O-GlcNAcylated serine/threonine-rich region of Sp1 destroys its association with heterotrimeric transcription factor NF-YA.^209^ The increased O-GlcNAcylation level significantly interrupts the interaction between coactivator Pgc1α and transcription factor PPARγ.^210^ In order to promote transcription, the N-terminal region of STAT5 was identified to be glycosylated to bind the coactivator of transcription CBP.^211^ The host cell factor C1 (HCF-1) was reported to recruit OGT to O-GlcNAcylate PGC-1α. This modification increased its interaction with deubiquitinase BAP1 to protect PGC-1α from degradation, thus facilitating downstream transcriptional activation.^212^ In another example, O-GlcNAcylation of the retinoblastoma-susceptibility gene product (pRB) promoted the binding and inhibition of f transcription factor E2F1.^213^

### Cancer signal cascade

Ketone bodies (β-hydroxybutyrate, acetoacetate, and acetone) serves as a metabolic regulator in glucose utilization, alanine, and non-esterified fatty acid supply, participating in histone PTM in many diseases, including cancer.^214,215^ β-hydroxybutyrate (BHB) and acetoacetate are the major ketone bodies so that it is important to study BHB-mediated post-translational modification in cancer. The intermediate product β-hydroxybutyrate has been reported to increase “stemness”-associated gene transcription for promoting tumor growth and metastasis.^216^ The p53 has long been studied in human cancer as a multifunctional transcription factor in the regulation of cell-cycle arrest, proliferation, senescence, aging, apoptosis, autophagy, ferroptosis, cell metabolism, and oxidative balance maintenance.^217–224^ In addition to histones, the only nonhistone protein with β-hydroxybutyrylation modification is p53. This modification occurs at sites 120, 319, and 370 of p53 lysine after the induction of BHB and is catalyzed by CBP/P300. The acetylation of p53 was reduced after β-hydroxyisobutyrylation, leading to inhibited p53 activation, weakened cell growth arrest, and apoptosis.^225^ Moreover, β-hydroxybutyration of p53 reduced its acetylation and expression of downstream factors p21 and PUMA, inhibiting cell growth arrest and apoptosis-mediated functions.^225^

**Fig. 3:**
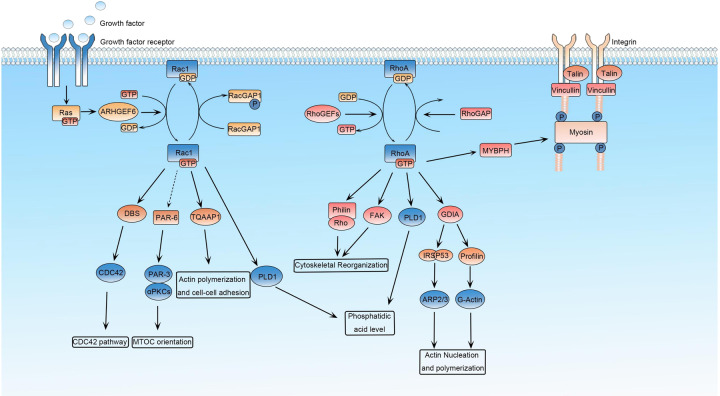
Ras is a membrane-associated guanine-nucleotide-binding protein that is normally activated in response to the binding of growth factors. Rac1 is a small G-protein in the Rho family that drives MTOC orientation, actin polymerization, and cell–cell adhesion. Rac1 is activated by ARHGEF6 and repressed by RacGAP in response to upstream regulators such as growth factors. Rho is a member of the Ras superfamily of small GTP-binding proteins that play a central role in diverse biological processes. Rho proteins cycle between an active GTP-bound state and an inactive GDP-bound state, which is controlled by regulatory proteins such as GEFs (guanine exchange factors and GAPs (GTPase-activating proteins). The GTPase RhoA plays a prominent role in regulating the organization of the cytoskeleton by promoting the assembly of focal adhesions and actin stress fibers and by activating FAK. PLD1 catalyzes the hydrolysis of phosphatidylcholine to yield phosphatidic acid and choline. Rho also activates scaffolding proteins such as GDIA and IRSp53 (insulin receptor substrate protein-53). RhoA also binds to Rho/philin and regulate the actin cytoskeleton. The Rho family members Rac1, RhoA, and CDC42 were reported geranylgeranylated, and Ras was reported farnesylated to regulate their cellular functions

It was found that the lysine at position 222 of lactate dehydrogenase A (LDHA) was recognized and highly succinylated by succinyltransferase CPT1A. The stability of succinylated LDHA was maintained to promote cell growth and invasion by reducing the degradation of ubiquitinated LDHA.^226^ Glutamine (Gln) can be converted to α- Ketoglutarate enters the tricarboxylic acid (TCA) cycle and plays a key role in synthesizing intermediates of mitochondrial metabolism, maintaining tumor cell growth, and signal regulation.^227^ The two glutaminase isoforms are liver-type (GLS2) and kidney-type (GLS), in which the site 311 of GLS is activated by succinylation to resist oxidative stress and promote tumor survival by increasing glutamine decomposition and NADPH/glutathione production.^228^ The growth of renal clear cell carcinoma can be regulated by the interaction between succinate dehydrogenase complex subunit A (SDHA) and SIRT5 and the level of succinylation.^229^ Many small G-proteins, including Ras, Rho, and Rac families, are prenylated with farnesyl or geranylgeranyl groups to help their membrane location. Several kinds of GTP-binding proteins acting as a molecular switch between active GTP-bound and inactive GDP-bound state are called Rho GTPase. The major function of Rho GTPases is the regulation of actin cytoskeleton and various corresponding cellular processes like cytokinesis, phagocytosis, pinocytosis, cell migration, morphogenesis, and axon guidance.^230^ In addition, the GTPase also regulate several biological pathways, including transcription factor nuclear factor κB (NF-κB), c-Jun amino-terminal kinases (JNKs) and mitogen-activated protein kinases (MAPKs) pathways, the phagocytic NADPH oxidase complex, G1 cell-cycle progression, the assembly of cadherin containing cell–cell contacts, polarized growth and mitogen-activated protein morphogenesis.^231–236^ The Rho family members Rac1, RhoA, and CDC42 were reported to be geranylgeranylated and Ras was reported to be farnesylated.^237^ (Fig. 3) Apoptosis may induce mitochondrial membrane permeabilization (MMP) to release catabolic hydrolases and activators or other proapoptotic molecules, leading to cell death.^238,239^ The most important regulators of apoptosis are the Bcl-2 protein family, which contains opposite function of antiapoptotic Bcl-2-like proteins (Bcl-2, Bcl-xL, Bcl-w, Mcl-1, and A1/Bfl-1), proapoptotic Bax-like proteins (Bax, Bak, and Bok/Mtd), and proapoptotic BH3-only proteins (Bid, Bim/Bod, Bad, Bmf, Bik/Nbk, Blk, Noxa, Puma/Bbc3, and Hrk/DP5).^240,241^ High levels of Bcl-2, Bcl-XL, and Mcl-1 were observed in multiple myeloma (MM) and responsible for cell viability and drug resistance.^242,243^ A post-translational geranylgeranyl lipid modification in C-terminal is essential for anchoring GTPase in the membrane. Other biological functions are found with specific PTM inhibitors. The enzyme 3-hydroxy-3-methylglutaryl coenzyme A (HMG-CoA) inhibitor lovastatin and geranylgeranyltransferase inhibitor (GGTI-298) caused dose-dependent apoptosis in human umbilical vein endothelial cells (HUVECs) through the activation of p53 and other proapoptotic proteins.^244^ In another study with myeloma cell lines and patient samples, the addition of GGTI-298 induced apoptosis through the downregulation of Mcl-1 protein expression, the disruption of the mitochondrial transmembrane potential, and cytochrome c release.^237^ The same results were observed in lymphoma cell lines and purified tumor cells from lymphoma patients after the inhibition of prenylation by lovastatin, the farnesyltransferase inhibitor (FTI-277), and GGTI-298.^245^ In adult T-cell leukemia (ATL), protein geranylgeranylation inhibition suggested anti-proliferative and apoptotic effects.^246^ Therefore, protein prenylation is a key event in the regulation of cancer cell survival. Ras protein family, including K-Ras, H-Ras, and N-Ras, are activated after GTP binding to regulate signal transduction from the extracellular environment to intracellular hemostasis. Ras activates several downstream signal pathways including Raf-MEK-ERK cascade, PI3K/Akt, and MAPK pathways.^247–250^ A high frequency of Ras mutation occurs in human cancer with suppressed Ras GTPase activity and constant GTP-binding state.^251^ This results in permanent signal transduction, leading to tumor cell growth, apoptosis, metabolism, and other biological processes.^252^ Lipid modifications are commonly occurred in the C-terminal of Ras proteins of yeast and mammalian cells, causing the attachment of Ras proteins with inner surface of the plasma membrane.^253^ Protein farnesylation occurs on the C-terminal CAAX motif to remove -AAX amino acids and leave C-terminal cysteine (Cys186). Studies found all Ras proteins undergone polyisoprenylation for their membrane association and function activation during human malignancy.^254,255^

The NMT proteins are responsible for protein myristoylation function in various cellular processes in human cancer. The N-terminal myristoylation is essential for the membrane attachment and kinase activity of Src-family protein tyrosine kinases (SFKs, including c-Src, Yes, and Fyn), which transduce signals to control cellular processes such as cell proliferation, adhesion, and motility.^256^ Early studies identified the positive influence of myristoylation on c-Src kinase activity and cellular transformation in contrast to the case for c-Abl.^257,258^ The myristoylated Src identified in human colon adenocarcinoma tumor promoted colony formation and cell proliferation.^259^ The inhibitor of NMT1 block Src myristoylation and cytoplasmic membrane location, as well as the inhibition of cell proliferation, migration, and invasion in prostate cancer cells and the inhibition of tumor growth in vivo.^260^ In addition to inhibitors, the depletion of NMT isozymes inhibited cell replication, induced apoptosis through regulation BCL family protein level and growth inhibition in vivo.^261^

The evolutionarily conserved protein p53 is the most intensively studied tumor suppressor in growth arrest and apoptosis and is regulated by a large number of proteins and PTMs. The MDM2 belongs to E3-ubiquitin ligase and promotes p53 ubiquitination and proteasome-dependent degradation.^262,263^ Since MDM2 was observed to be conjugated to NEDD8, the neddylated p53 was also detected in vitro and in vivo, with the dependence on MDM2-p53 interaction. MDM2-mediated neddylation of p53 suppressed its transcriptional activity.^189^ The mRNA binding and stabilization protein Hu antigen R (HuR) is another neddylation substrate of MDM2. High expression of HuR was tested in several cancer types to regulate proliferation, differentiation, transformation. In the studies of colon cancer and hepatocellular carcinoma (HCC), MDM2-mediated HuR neddylation at sites K313 and K326 promoted its nuclear localization, avoiding cytoplasmic ubiquitination and degradation.^264,265^ The higher expression level of neddylation enzymes NAE1 and UBA3 in glioblastoma with poor clinical outcomes indicated neddylation can be considered as a therapeutic target. In glioblastoma cell lines and patient-derived glioblastoma stem cells, the neddylation inhibition by inhibitor MLN4924-induced apoptosis, which was associated with activation of the ERK-MAPK and PI3K-AKT signaling pathways.^266^

The pathogenic function of NO in tumor depends not only on the direct promotion or inhibition effects of nitrosative and oxidative stress, DNA damage-induced RNS formation, mitochondrial dysfunction, and apoptosis but also on the regulatory effect of protein S-nitrosylation.^267^ For example, S-nitrosylation may inhibit kinase or phosphatase activity. The apoptosis signal-regulating kinase 1 (ASK1, also MEKK5) is associated with cell apoptosis and mitochondrial damage under cellular stress in p38 kinase-dependent way and Hippo signaling effectors YAP/TAZ mediated cell invasion/migration.^268^ The interferon-gamma (IFN-γ) induced NO production and S-nitrosylation of ASK1, blocking the interaction of ASK1 with its downstream effectors MKK3 or MKK6.^269^ Moreover, IFN-γ promoted endogenous S-nitrosylation of JNK activation and disrupted JNK and its substrate c-Jun interaction.^270,271^ PTEN (phosphatase and tensin homolog) is a tumor suppressor controls a variety of biological processes. PTEN antagonizes the PI3K/Akt signaling pathway and can be regulated by several kinds of PTMs such as ubiquitylation, SUMOylation, neddylation, S-nitrosylation, and phosphorylation.^272,273^ Cys-83 is the S-nitrosylation site of PTEN under the condition of low NO concentrations and Akt signal is promoted after PTEN S-nitrosylation.^274^

The function of O-GlcNAcylation in human cancer is also confirmed by the studies about O-GlcNAcase. Primary breast tumor tissues showed an increased O-GlcNAcase and lysosomal hexosaminidase activity than the corresponding adjacent normal samples, with a significantly decreased O-GlcNAc mono-glycosylation.^275^ The enzyme activity of O-GlcNAcase was also increased in thyroid cancers and the modified proteins showed a predominantly nuclear distribution.^276^ This situation is different in other studies. Researchers identified enhanced GlcNAcylation level in breast tumor as compared to normal samples, and even higher in metastatic lymph nodes. Cell migration and invasion were suppressed after lentiviral-mediated OGT silencing and increased in OGA-specific inhibitors NButGT and PUGNAc treatment.^277^ In the study of lung and colon cancer, not only O-GlcNAcylation and global O-GlcNAc transferase expression but also the OGT mRNA levels were evaluated in tumor tissues.^278^ Similarly, a high level of O-GlcNAcylation in target proteins was detected and was associated with the pathogenesis of characterizes chronic lymphocytic leukemia.^279^ O-GlcNAcylation targets are involved in various biological processes and signal cascades. O-glycosylated carboxy-terminus of p53 increases DNA binding.^280^ The eukaryotic translation initiation factor 2 (eIF2) is one of the widely studied kinase regulating cell growth, differentiation, metabolism, and stress response, which was reported to be regulated by several PTMs.^281^ Glycosylation of p67 protein is required to protect the eIF2 alpha-subunit from eIF2 kinase phosphorylation.^282^ O-GlcNAcylation of mammalian neurofilament-H subunits appears to regulate intermediate filament network formation in large myelinated neurons.^283^ Considering the central role of O-GlcNAcylation in the regulation of protein interaction and gene transcription, it is reasonable to raise questions on the occurrence and function of O-GlcNAcylation in the cancer signal pathway. Wnt/β-catenin signaling cascade is conserved and has emerged as a fundamental growth and development regulation pathway. Mutation of WNT/β-catenin signaling is frequent in many human cancers.^284^ Nuclear accumulation of β-catenin enhances the interaction with transcription factors T-cell factor (TCF)/lymphoid enhancer factor (LEF) to activate Wnt target genes. O-GlcNAcylation of β-catenin at Ser23 increased the movement of β-catenin from the nucleus to the plasma membrane, promoted the interaction of β-catenin with E-cadherin, and decreased transcription activity of Wnt signal.^285^ O-GlcNAcylation of β-catenin at T41 showed a direct competition with phosphorylation and a promotion of α-catenin–β-catenin interaction, which is critical for mucosa integrity.^286^ FoxM1 is an important transcription factor regulating cell proliferation, differentiation, transformation, cell cycle, and cell invasion in many types of cancer.^287–290^ Inhibition of the high level of OGT and O-GlcNAc in breast cancer cells reduced expression of FoxM1 and its target proteins, leading to the inhibition of breast cancer phenotypes.^291^ As one of the most critical transcription regulators in normal and tumor cells, c-Myc was also reported to be modified by O-GlcNAcylation even in the same site of phosphorylation.^292,293^ PTM of this site may regulate transforming activity and tumorigenicity in lymphomas.^294^ P53 is a crucial tumor suppressor and its fundamental biological function in tumorigenesis and cancer development is well studied in the regulation of genome instability, transcription, cell-cycle arrest, apoptosis, cellular metabolism as well as autophagy. Researches about the vast regulation network of p53 ranges from gene polymorphisms, transcription, PTM, protein–protein interaction to degradation.^295^ Besides the regulation by the E3-ligase oncoprotein MDM2 in p53 protein activity and degradation, the cellular function of p53 is also affected by PTM. P53 undergoes O-GlcNAcylation at site Ser149, thus inhibits its phosphorylation at Thr155. This modification abrogates the ubiquitination-dependent proteasomal degradation and p53–MDM2 interactions.^296^ Kelch ECH-associating protein 1 (Keap1) is a substrate for the Cullin-3 (CUL3)-dependent E3-ubiquitin ligase complex regulating the transcription factor erythroid 2-related factor 2 (Nrf2) to keep intracellular homeostasis under stress condition.^297^ With the help of a chemical biology approach in MDA-MB-231 cells, O-GlcNAcylation in Keap1 was identified at site Ser104 to promote CUL3 interaction and play a role in ubiquitination and degradation of Nrf2.^298^

Multiple lipid modifications, including isoprenylation, myristoylation, palmitoylation, and glycosylphosphatidylinositol anchor, may lead to distinct protein subcellular location and various signal transduction.^299^ The concept of apoptosis, a programmed cell death process, is essential for homeostasis maintenance.^300^ It was reported that N-terminus myristoylation is due directly to caspase cleavage and autophagy.^301^ Sirtuins are well known for their lysine deacetylases activity to regulate biological processes. Besides the efficient deacetylase function, SIRT1–3 have been recently identified to remove long-chain fatty acyl groups. Among them, SIRT2 prefers more efficient demyristoylase activity.^302^ The tumor suppressor SIRT6 is also reported to remove fatty acyl groups from the lysine residues from R-Ras2. Lipid-modified R-Ras2 produces a marked oncogene effect including tumor growth, signal transduction, cytoskeletal dynamics, and PI3K-associated cell proliferation.^303^

### Metabolic regulation

Metabolic reprogramming is a hallmark of cancer cells. A rapid synthesis of structure blocking molecules including nucleotides, proteins, and lipids is necessary to support fast cancer cell growth. Under this condition, cancer cells exhibit increased glucose uptake, as well as fatty acid synthesis and glutamine consumption.^304,305^ Many metabolic enzymes are reported abnormal in tumorigenesis and cancer development. In order to meet the lipid demand of rapid proliferation, the enzymes related to de novo lipid synthesis in tumor cells are abnormally highly expressed, such as fatty acid synthase (FASN) which catalyzes the synthesis of palmitic acid by acetyl-CoA and malonyl-CoA.^306,307^ One of the possible mechanisms of upregulated FASN under hypoxia of TME is the activation of Akt-HIF-1 axis and the following induction of transcription factor SREBP-1.^308^ Multiple acetylation sites were identified on FASN. Acetyltransferase KAT8 mediates ubiquitin–proteasome-related FASN degradation and the deacetylation depends on HDAC3n.^309^ Therefore, targeting FASN acetylation can be used as a new direction of tumor therapy. Cancer cells expressed a splice isoform of the glycolytic enzyme pyruvate kinase (M2), which was necessary for tumor formation in nude mouse xenografts.^310^ During Akt-mediated tumorigenesis, the conversion of glucose to lipid depends on the activity of ATP citrate lyase (ACL), which promotes the production of cytosolic acetyl-CoA from mitochondria-derived citrate.^311^ Conversely, mutations in some oncogenes can affect cancer-associated metabolic reprogramming. Increased expression of the serine/threonine kinase Akt raises glucose consumption to support aerobic glycolysis and tumor survival.^312^ Increased levels of glycolysis, glutamine metabolism, and nucleotide biosynthesis were also observed after oncogene KEAS expression.^313^

Multiple CoA generated from the TCA cycle, as well as the metabolism of amino acid and lipids can be the substrates of PTM such as malonyl-CoA, succinyl-CoA, and glutaryl-CoA, highlighting their emerging role in metabolic regulation. Malonyl-CoA is the two-carbon donor in de novo fatty acid biosynthesis and fatty acid elongation, and also the inhibition of carnitine palmitoyltransferase 1 (CPT1) in mitochondrial β-oxidation, hepatic fatty acid synthesis, and ketogenesis.^314,315^ In mammalian cells, the ACS family member ACSF3 localizes to the mitochondrial matrix and is required for lysine malonylation of mitochondrial proteins.^316^ Malonyl-CoA can be converted to acetyl-CoA by malonyl-CoA decarboxylase (MCD), which mutations cause an inborn metabolic disorder.^317^ With affinity enrichment of malonylated peptides, MCD-deficient cells, and SIRT5 KO mice showed increased lysine malonylation, as well as impaired mitochondrial respiration and fatty acid oxidation.^318^ By the characterization of succinylation proteome, succinylated proteins are discovered significantly enriched in cellular metabolic process, including oxoacid metabolism, oxidation–reduction process, and coenzyme metabolism.^319^ As previously mentioned, Sirt5 functions in lysine demalonylation and desuccinylation, which has been identified to regulate metabolic proteins. The mitochondrial pyruvate dehydrogenase complex (PDC) catalyzes decarboxylation of pyruvate to acetyl-CoA, linking glycolysis to the TCA cycle.^320^ In the depletion of SIRT5 in MEF cells, multiple subunits of PDC were hypersuccinylated, suggesting the negative regulation of SIRT5 in PDC activity.^319^ Succinylation of mitochondrial proteins will affect their normal function and redox state to ensure that cells respond quickly under environmental changes in TME with abnormal metabolic programming.^321^ Pyruvate kinase PKM2 shows the transcription factor property and enzymatic activity of promoting tumor aerobic glycolysis.^322^ The activity of PKM2 was enhanced by succinylation at K498. ROS promotes the binding and desuccinylation of PKM2 by SIRT5, reducing NADPH production, so as to achieve growth inhibition.^319,323^ When the nutritional environment changes such as glucose starvation, the succinylation of PKM2 is involved in regulating its mitochondrial translocation and coordinating cell proliferation or survival.^324^

Myristoylation and palmitoylation participate in nutrient absorption and cell metabolism by regulating mitochondrial function. AMP-activated protein kinase (AMPK) maintains cellular ATP homeostasis, senses intracellular energy level, and is activated by metabolic stimulation.^325,326^ Under the starvation condition, cell relies on autophagy to provide energy, and this process is regulated by AMPK.^327^ Autophagy and impaired mitochondrial clearance in AMPK deficient cells are hindered. Myristoylated AMPK enhances the connection with mitochondria through membrane fusion and maintains the function of AMPK mediated autophagy.^328^ Fatty acid metabolism is an important step in metabolic reprogramming of tumor cells, which comes from exogenous uptake or de novo synthesis of other metabolic intermediates (glucose or glutamine). Membrane protein CD36 is a fatty acid translocase (FAT) and its palmitoylation can regulate the subcellular distribution and function of proteins.^329^ The palmitoylation and plasma membrane localization, as well as fatty acid uptake of CD36, require DHHC (Asp–His–His–Cys) motif-containing palmitoyl acyltransferases, DHHC4 and DHHC5.^330^ Palmitoylation of a coding product of the oncogene KRAS (KRAS4A) determines its interaction with hexokinase 1 (HK-1) and outer mitochondrial membrane (OMM) localization, thereby regulating kinase activity and glycolytic flux.^331^ Another important oncogene, epidermal growth factor receptor (EGFR), can also be regulated by palmitoylation. The stimulation of palmitic acid de novo synthesis by plasma membrane EGFR (pmEGFR) can promote the palmitoylation of mitochondrial EGFR (mtEGFR), and then promote mitochondrial fusion and tumor cell growth.^332^ This mechanism extends the understanding of EGFR to promote cancer progression independent of its tyrosine kinase activity. Mevalonate (MVA) is a common building block of many cellular compounds, including the membrane structure and isoprenoid/cholesterol biosynthetic precursor cholesterol,^333^ the N-linked glycoproteins carrier phosphorylated dolichol,^334^ the electron transfer lipid in the mitochondrial respiratory chain ubiquinone (UQ, coenzyme Q),^335^ some tRNAs modification protein substrate isopentenyladenine and the substrates geranylgeranyl pyrophosphate (GGPP) and farnesyl pyrophosphate (FPP) for prenylation.^336^ HMG-CoA catalyzes the conversion of HMG-CoA to MVA, which is inhibit by lovastatin. Lovastatin is an effective anticancer drug in many types of cancer like acute myelogenous leukemia, medulloblastoma, mesothelioma and neuroblastoma.^337–340^ The same apoptotic effect of lovastatin was also observed in GGTI-298 and less effective in the FTI-277, indicating the role of prenylation, especially geranylgeranylation, in lovastatin-induced apoptosis in AML.^341^

In recent years, a series of novel functions of lactate have been discovered such as the fuel for metabolic processes, the promotion role of tumor invasion and metastasis, the function in angiogenesis and especially, the immune suppression in tumor microenvironment. Lactate can be absorbed by cancer cells and transported to mitochondria for oxidation to provide energy. Lactate in tumor microenvironment can inhibit the cytotoxicity of immune cells, suggesting an essential role of lactate in metabolism regulation. Warburg effect in cancer cells explains the lactate formation from glucose. Lactate can also be produced from glutamine metabolism. Glutamine is catalyzed by glutaminase GLS to transform into glutamate, and glutamate dehydrogenase convers glutamate intoαKG to enter TCA cycle.^114^ In some types of cancers like human pancreatic ductal adenocarcinoma (PDAC), glutamate and oxaloacetate (OAA) are transformed into αKG and aspartate. The aspartate can be converted into oxaloacetate by aspartate transaminase GOT1, followed by the conversion of oxaloacetate to pyruvate.^342^ Finally, the enzyme LDHA catalyzes the conversion of pyruvate to lactate.^114^ Lactate transport into cells is mediated by monocarboxylate transporters (MCTs) family proteins MCT1 (also SLC16a1), MCT2 (also SLC16a7), MCT3 (also SLC16a8), and MCT4 (also SLC16a3).^343^ Since first identified, the lactate-derived lysine lactylation is found associated with cellular metabolism. Glucose can induce lactate production and histone lactylation in a dose-dependent manner and metabolic labeling experiments proved lysine lactylation is endogenously derived from glucose.^120^ Lactic acid stimulated the increased levels of histone lactation in HK-1 and IDH promoters in NSCLC and breast cancer cells.^344^ In addition, the transcription of HK-1, G6PD, and PKM was downregulated, while the transcription of SDH, IDH, and HIF1A was upregulated.^345^ This supports that lactic acid regulates the gene expression of related metabolic enzymes through histone lactylation, so that tumor cells show different metabolic characteristics.

The high-energy nucleotide sugar uridine diphosphate-GlcNAc (UDP-GlcNAc) derived from glucose and other nutrition metabolites is required for protein O-GlcNAcylation.^346^ The biochemical and phenotypic influence of O-GlcNAcylation is implicated on metabolic disorders and human diseases, particularly cancer. In response to nutrient levels, O-GlcNAcylation was reported to regulate signaling cascade, protein solubility and stability, gene transcription, and genome replication.^347^ In one study of basal-like breast cancer, a high level of O-GlcNAcylation and α-ketoglutarate-dependent transcription factor HIF-1α, as well as its downstream factor GLUT1, were observed. Mechanistically, changed OGT level regulated HIF-1α proteasomal-dependent degradation, the interaction between HIF-1α and pVHL, ER stress-mediated tumor cell survival, and cell metabolism.^348^

The newly identified link between neddylation modification with metabolism is studied by neddylation inhibitor MLN4924. As an energy hub in metabolism, mitochondria function, morphology, and fusion-fission dynamics should be fine-tuned.^349^ Under the condition of oncogene signaling, hypoxia environment, nutrient starvation and anticancer treatment, mitochondria maintain the balance between rounded, fragmented signature with elongated, interconnected shape to regulate its cytoskeletal transport activity and metabolic function. Abnormal mitochondrial regulation contributes to cancer hallmarks, including cellular transformation, cell survival in therapy response, cancer stem cell maintenance, cell differentiation, and migration. Several mammalian mitochondrial proteins undergo PTMs to keep mitochondrial homeostasis.^350^ Through a global metabolic profiling analysis after the treatment of MLN4924, the nucleotide biosynthesis pathway was disrupted in AML cells.^351^ A recent study connects altered energy metabolism with protein neddylation. MLN4924 may induce autophagy partially through the inhibition of mTOR activity and induction of ROS stress in liver cancer cells.^352^ Moreover, mitochondrial fission-to-fusion conversion was induced by MLN4924 treatment in breast cancer cells. The overall metabolism, including carbohydrates, organic acids, amino acids, nucleotides, and lipids were inhibited. As for the altered metabolites, the increased 3-phosphoglyceric acid and pyruvic acid, as well as the decreased succinic acid and fumaric acid implied enhanced glycolysis, reduced mitochondrial functions, increased mtDNA copy number, and mitochondrial respiration (OXPHOS).^353^ Therefore, the combination strategy of neddylation inhibitor and metabolism regulation agents is the new avenue to enhance anticancer efficiency.

### Epithelial–mesenchymal transition (EMT) regulation

**Fig. 4:**
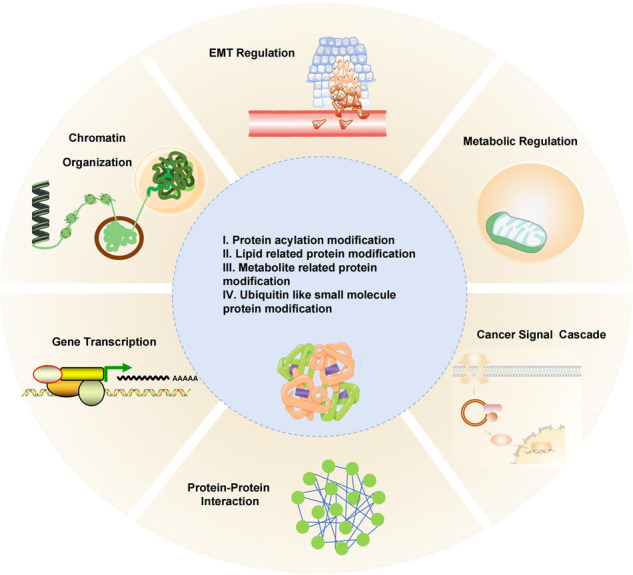
Biological effect of modification on tumor initiation and development. Five groups of PTMs function in cellular biological processes, including altering higher-order chromatin structures to positively or negatively regulate gene transcription, modulating protein attachment on membrane or transcription factors to regulate downstream signal transduction, changing metabolic enzymes and related transcription factors to regulate metabolic reprogramming, as well as remodeling cytoskeleton and cell adhesion system to regulate tumor invasion

Protein post-translational modification participates in all stages of cellular processes (Fig. 4). The interaction between different cells and the attachment of one cell to its extracellular matrix are important for cell movement and tissue structural integrity. As extracellular vehicles (EVs) secreted by tumor and other cells in TME, exosome is involved in intercellular communication and regulate immune response by expressing histocompatibility complex MHC classes I and II.^354^ Different studies have found that tumor-derived exosomes can promote fibroblast differentiation, contribute to tumor metastasis and participate in TME reprogramming in a TGFβ dependent manner.^355,356^ A melanoma metastasis model proved that the release of anti-metastatic EVs depends on the acetylation of p53, and BAG6/CBP/P300 is essential in this process.^357^ The biomechanical properties of tumor cells in TME change with extracellular matrix (ECM) and intracellular cytoskeleton structure to enhance the possibility of invasion.^358^ The cytoskeleton mainly composed of microtubules and actin not only maintains the basic morphology of cells but also participates in the localization and transportation of organelles.^359^ Interestingly, the cytoskeleton proteins are often suffered a variety of PTM modifications, including phosphorylation, ubiquitination, palmitoylation, glycosylation, and acetylation.^360^ Lysine at position 40 of α-tubulin is acetylated by acetyltransferase αTAT1 and this process is reversed by deacetylases HDAC6 and SIRT2.^361,362^ The high acetylation of α-tubulin is associated with the proliferation and invasive activity of breast cancer and colon cancer, which can be used as a potential marker.^363,364^ Disruption of microtubule acetylation can cause the increased endoplasmic reticulum stress signal to inhibit tumor progression.^365^

The S100 protein family show widely functions in every stage of cancer development by regulating key signal pathways.^366^ One of the membrane-located S100A10 was reported highly expressed in ovarian cancer, breast cancer, colorectal cancer, renal cell carcinoma, and gastric cancer.^367–371^ The high level of succinylation of S100A10 at K47 was found in gastric cancer and metastatic lymph node tissue to inhibited ubiquitination-related proteasome degradation. Mechanically, this succinylation is mediated by succinyltransferase CPT1A to promote cell migration and invasion.^372^

Proteins that participate in cell adhesion system can be divided into three types: cell adhesion molecules (cadherins, integrins, selectins, and immunoglobulins), the highly organized and dynamic three-dimensional (3D) scaffolds ECM (including collagens, fibronectins, laminins, and proteoglycans) and membrane proteins located at cell peripheral, with a linkage function of adhesion systems to the cytoskeleton.^373–375^ These proteins also regulate signal transduction, membrane trafficking, and cell division.^376^ Cadherin family members include the epithelia expressed E-cadherin, the neural and mesenchymal tissue expressed N-cadherin, and vascular endothelia-specific VE-cadherin.^377^ The various cadherin superfamily proteins function as oncogenic or tumor suppressor to affect tumorigenesis and/or tumor progression, making these cadherins potential therapeutic targets for cancer. A current research theme is the various cadherins regulation and cadherin switch during EMT and cancer progression.^378^ The Rho family of small GTPases, especially Rho, Rac, and Cdc42, were demonstrated to regulate cadherin-mediated adhesion, and thus EMT process.^379–382^ Ras family also belongs to small GTPases and one of the members Rap1 (with the isoform Rap1A) is a major activator of integrins to regulate cellular functions and cell adhesion.^383^ Activation of Rap1 promotes prostate cancer survival and metastasis.^384^ The inhibition of protein geranylgeranylation, but not protein farnesylation, reduced Rap1A and Rab6 geranylgeranylation and prevented prostate cancer metastasis.^385^ The result shed light on the connection between protein geranylgeranylation with cancer metastasis.

The regulation of O-GlcNAcylation in cancer is also reflected in the EMT process. The snail family member snail1 was reported to induce EMT by directly repressing the transcription of E-cadherin.^386^ Glycogen synthase kinase 3β (GSK3β) and protein kinase CK1 are responsible for binding and phosphorylating Snail to regulate beta-Trcp-mediated ubiquitination and subcellular localization.^387,388^ Cancer cells have to break down cell–cell and cell–matrix attachment in EMT, which involves cadherin switch, intrinsic and extrinsic signals, as well as a dramatic reorganization of the cytoskeleton. As a highly dynamic structure regulated by Rho GTPase family, cytoskeleton modulates cell motility and invasion through forming filopodia, lamellipodia, podosomes and invadopodia into the surrounding environment.^389^ E-cadherin is a single-span transmembrane glycoprotein and a major component of epithelial adheren junctions (AJ) to mediate cell–cell adhesion.^390^ Under the condition of ER stress-induced apoptosis, E-cadherin and β-catenin were O-glycosylated and led to reduced intercellular adhesion.^391^ Moreover, the phosphorylation-mediated Snail1 degradation is blocked by O-GlcNAcylation at site Ser112, leading to EMT stimulation through transcriptional suppression of E-cadherin.^392^

The NAE inhibitor MLN4924 revealed the metastatic regulation of protein neddylation. Liver metastatic veal melanoma (UM) patients showed highly expressed key proteins in the neddylation pathway. The MLN4924 treatment specifically decreased cullin1 neddylation, facilitated the accumulation of CRL substrates, induced mitochondrial damage, triggered intrinsic apoptosis, enhanced DNA double-strand breaks and ROS generation, diminished UM cells migration and invasion, and repressed the cancer stem cells (CSCs) properties through Slug protein degradation. The inhibit tumor outgrowth, disturbed paracrine secretion of VEGF-C, and impaired angiogenesis were observed in NOD-SCID mouse xenograft model.^393^ The inhibition of the metastatic process by MLN4924 was conformed recently in lung cancer. First, key proteins in neddylation process are overactivated in both lung adenocarcinoma and squamous cell carcinoma. Second, inhibition of neddylation using MLN4924 significantly suppressed cell growth, clone formation, migration, and cell motility in vitro, as well as tumor formation and metastasis in vivo. Lastly, phorbol-12-myristate-13-acetate-induced protein 1 (NOXA)-dependent apoptosis was induced by MLN4924.^394^ The anti-migration effect by activating E-cadherin and the anti-invasion effect repressing Vimentin were observed after MLN4924 treatment in human clear cell renal carcinoma (ccRCC).^395^ Mechanistically, MLN4924 inhibit intravascular survival, extravasation, and formation of metastatic colonies to inhibit lung cancer metastasis. At an early stage, MLN4924 downregulated the expression of MMP2, MMP9, and vimentin to disrupt actin cytoskeleton. At a later stage, MLN4924 block neddylation to increase its substrates like p21, p27, and Wee1, leading to cell-cycle arrest and apoptosis.^396^

## PTM-dependent immune surveillance and escape in TME

### Acidic environment

Cancer cells export metabolic waste such as lactate into TME to form an acidic environment. The low pH might influence the modification of proteins both in tumor cells, immune cells, fibroblast cells and so on. In addition, low pH is closely corrected with hypoxia and low blood-flow levels, as well as the effectiveness of various therapeutic agents.^397^

### Hypoxia environment

In the inner tumor microenvironment, the concentration of oxygen is much lower and form a condition called hypoxia. The increased oxygen consumption caused by high rate of tumor cell proliferation and decreased oxygen supply caused by compact tumor microvasculature network are main reason of hypoxia formation.^398,399^ Under conditions of reduced oxygen, hypoxia-inducible factor 1 (HIF-1) is activated, leading to the expression of vascular endothelial growth factor (VEGF) to stimulate angiogenesis.^400,401^ Up to 95% of FA in tumor cells is de novo synthesis, and the intermediate metabolite palmitic acid is produced from acetyl-CoA, malonyl-CoA, and NADPH through catalysis by FASN.^402^ In vivo studies found that the angiogenesis of FASN knockout mice was impaired. The depletion of FASN enhanced the accumulation of malonyl-CoA and reduced mTOR activity through mTOR malonylation.^403^

More and more regulatory functions of HIFα in cancer progression are revealed including the maintenance of cancer stem cells by regulation of Notch pathway,^404^ the negative regulation of mitochondrial biogenesis and O_2_ consumption,^405^ the regulation of glucose metabolism by interacting with pyruvate kinase isoforms PKM2,^406^ the induction of wild-type EGFR overexpression,^407^ the suppression of E-cadherin,^408^ the promotion of metastatic cascade,^409,410^ and the resistance to radiotherapy and chemotherapy.^411,412^ Hypoxia-mediated O_2_ homeostasis plays an important role in the pathogenesis of major causes of mortality, especially in cancer. The expression of some glycolytic enzymes is induced by the transcriptional oncoproteins HIF-1α, reflecting the close correlation of hypoxia microenvironment and cellular metabolism. These enzymes include hexokinase 2 (HK2), glucose-6-phosphate isomerase, 6-phosphofructokinase 1, aldolase, fructose bisphosphate (FBP), triose phosphate isomerase (TPI), phosphoglycerate kinase (PGK1), enolase 1, PKM, and LDHA.^114,413–415^ In addition, HIF-1 upregulates PD-L1 expression in immune cells like MDSCs and macrophages, constructing an immunosuppressive tumor environment.^416–418^ Another checkpoint VISTA (V-domain Ig-containing suppressor of T-cell activation) is also overexpressed under hypoxia condition, especially in the immunosuppressive MDSC cells.^419^ In the phagocytosis function of macrophage, cell surface protein cluster of differentiation 47 (CD47) binds with its ligands, signal regulatory protein α (SIRPα), and thrombospondin-1 (TSP-1), to conduct a “don’t eat me” signal, facilitating tumor cells escape.^420,421^ Future studies targeting selective inhibitors of hypoxia have a large potential in cancer drug discovery.

### Immunosuppression effect

Recently, more and more studies reported the functions of metabolic production and the related protein PTMs in impairing the adaptive immune response or disabling immune surveillance. We discuss the immunosuppression function of several types of PTMs in this review.

#### Innate immune response and IFN signal

**Fig. 5:**
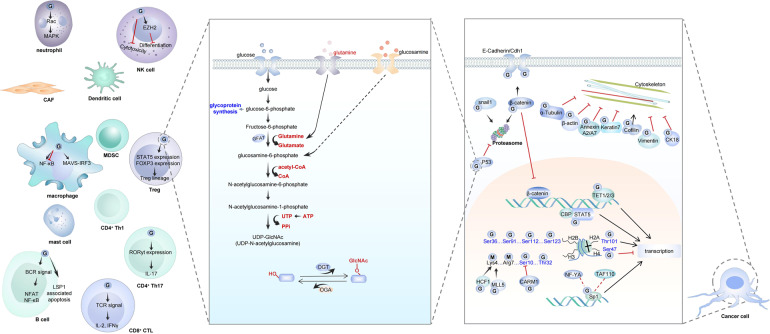
Overview of the hexosamine biosynthetic pathway (HBP) and the regulation of O-GlcNAcylation in TME. Left: O-GlcNAcylation orchestrates immunity. O-GlcNAcylation is increased after neutrophils are activated through the MAPK pathway. In macrophages, O-GlcNAcylation shows both activation and inhibition functions of NF-κB. Upon RNA virus infection, MAVS is modified by OGT to enhance downstream IFN production via IRF3 signaling. O-GlcNAcylation reduces the cytotoxic activity and inhibits NK differentiation by increasing the stability of EZH2. O-GlcNAcylation increases the expression of RORγt and FOXP3 in Th17 and Treg cells. NFAT and NFκB are activated by O-GlcNAcylation in activated B cells and O-GlcNAcylation of LSP1 contributes to B-cell apoptosis. After TCR rearrangement, O-GlcNAcylation promotes positive T-cell development. Middle: nutrient flux modulates protein O-GlcNAcylation. OGT catalyzes the addition of GlcNAc from UDP-GlcNAc to serine and threonine residues, while OGA catalyzes their removal. Right: the function of O-GlcNAcylation in proteasome-associated degradation, cytoskeleton remodeling, and transcriptional regulation

Innate immune response triggered by viral infection may activate transcription factors NF-κB and IRF3 to regulate the expression of type I interferon and interferon-stimulated genes (ISGs).^422^ After the RNA virus infection, they may replicate to produce double-stranded RNA. Protein RIG-1 and the downstream factor MAVS (mitochondrial antiviral signaling) are involved in recognizing dsRNA and producing interferon to initiate the innate immune response.^423,424^ The mitochondrial-associated membrane (MAM) links the endoplasmic reticulum to mitochondria, where RIG-1 interacts with MAVS to form an intracellular immune synapse^425^ (Fig. 5). In a recent study with bone marrow-derived macrophages (BMDMs), protein geranylgeranylation was observed to inhibit RIG-I-like receptor (RLR) mediated innate immune signaling pathways. Moreover, anchoring the Rho GTPase Rac1 into MAM needs protein geranylgeranylation and subsequent palmitoylation. MAM-associated Rac1 promotes MAVS signaling complex formation and then inhibits the interaction between MAVS and the E3 ligase Trim31, facilitating the association of caspase-8 and cFLIPL and MAVS signal termination.^426^

Pathogenic alteration produces host-derived intrinsic stimulation to induce inflammation. The cytokines IL-17A contribute to immune response in cancer and is and is secreted from IL-17A-producing CD4^+^ T cells (T_h_17 cells), CD8^+^ T cells, γδT cells, and natural killer T cells. IL-17A was reported to promote hepatoma stem cells self-renewal and immune escape partly through upregulation of PD-L1.^427^ T_h_17-produced IL-17A is induced by IL-1b and IL-23, which is enhanced by lactate.^428^ Lactate was reported to bind the complex of retinoic acid-inducible gene-I/mitochondrial antiviral-signaling protein (RIG-I/MAVS) transmembrane (TM) domain and inhibit MAVS aggregation, thus suppressing glycolysis-mediated RLR signaling and type I IFN pathway.^429^

Toll-like receptor (TLR) family has a crucial role in the field of innate immunity and also connects non-specific immunity and specific immunity, even individual TLRs exhibit specific responses.^430,431^ TLR4 colocalized with TRIF-related adaptor molecule (TRAM) in the plasma membrane and the Golgi apparatus to trigger an intracellular signaling cascade, including the production of IRF3 dependent type I IFN- and NF-κB-dependent pro-inflammatory cytokines. Interestingly, TRAM contains an N-terminal myristoylation sequence, and the myristoylated TRAM tethers it to the membrane, serving as a prerequisite for LPS signaling.^432^ Moreover, TRAM was reported to function in TLR7 signaling in the detection of single-stranded RNA (ssRNA) derived from RNA viruses. The myriostoylation of TRAM is necessary for TLR7 mediated CCL5, IFN-α, and IFN-β activation.^433^ These results supported the function of myristoylation in innate immune response mainly by its property in mediating protein location in the cell membrane.

#### Macrophage

Macrophages are large, mononuclear, highly phagocytic cells derived from monocytes and play a key role in innate immune response and pathogen defense. In addition to phagocytosis, macrophages can secrete a vast array of mediators including enzymes, inflammatory cytokines, growth factors, eicosanoids, and oxidants. The diverse cellular functions are regulated by specific microenvironments and inflammatory mediators.^434^ The type I cytokines like interferon-γ (IFNγ), tumor necrosis factor-α (TNFα), and pathogen-associated molecular patterns (PAMPs, including lipopolysaccharide (LPS), lipoproteins, dsRNA, lipoteichoic acid) activate macrophage and promote Th1 immune responses, whereas macrophage stimulated by Interleukin (IL)-4, IL-13, IL-1β, IL-10, transforming growth factor-β (TGFβ), and glucocorticoids support Th2-associated function.^435^ Toll-like receptors (TLRs) recognize microbial components and the best characterized is LPS.^436^ The interaction between LPS and TLR4 activates the molecules myeloid differentiation factor 88 (MyD88) or TIR-domain-containing adaptor inducing IFN-β associated signal pathways, leading to the phosphorylation and ubiquitin-mediated degradation of IκB proteins and the latter the activation of transcription factor NF-κB.^437^ The neddylation inhibitor MLN4924 and depletion of Nedd8 or the Nedd8-conjugating enzyme by siRNA are used to demonstrate the regulation of neddylation in macrophage function. The decreased neddylation process disrupted pro-inflammatory cytokines secretion from macrophages through the repression of NF-κB in response to LPS.^438^ BMDM stimulated by LPS can produce a large amount of malonyl-CoA and cytokines like IL-6 and TNF α. It was further confirmed that the substrate of malonylation induced by LPS was GAPDH at the site of lysine 213, which is essential for cytokine production.^439^ This confirms the role of PTM regulation targeting GAPDH in the inflammatory response. During LPS stimulation, a select group of macrophage proteins, including a protein kinase C (PKC) substrate, were identified to be myristoylated. This modification targeted protein location to the membrane to interact with PKC.^440^ Other substrates for PKC in macrophage were myristoylated by IFN-γ, but not IFN-α nor IFN-β, to mediate intracellular signal pathways.^441,442^ In *NMT1*^+/−^ mouse model, the depleted colony-forming ability of BMDM was observed, suggesting the role of protein myristoylation in monocytic differentiation.^443^ Macrophages undergo a metabolic reprogramming from pro-inflammatory “M1” macrophage with high phagocytic and bactericidal potential to “M2” macrophage with anti-parasitic and tissue repair functions.^444,445^ The low level of arginase-1 (ARG1) produces enhanced nitric oxide synthase (iNOS) to catalyze arginine to citrulline and nitric oxide and then kill pathogens in “M1” macrophage. On the other hand, ARG1 is induced to produce urea, polyamines, and ornithine in “M2” macrophage.^446,447^ During “M1” macrophage activation, lactate and the corresponding histone lactylation plays a positive role in driving expression of M2-like genes, reflecting a “lactate clock” in gene expression regulation.^120^

#### TCR signal

**Fig. 6:**
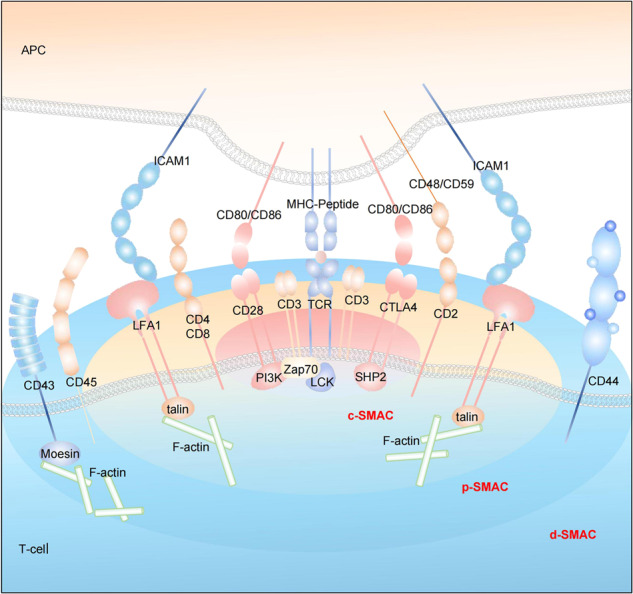
Overview of a mature T-cell synapse. The immunological synapses (IS) is also termed supramolecular activation complexes (SMACs), including the central region of the supramolecular activation complex (c-SMAC), the peripheral ring surrounding the c-SMAC (p-SMAC), the region distal to the synapse outside the p-SMAC (d-SMAC) and the molecules/ligand pairs that are found enriched within. Protein myristoylation makes a significant contribution to IS formation. APC antigen-presenting cell, CTLA4 cytotoxic T-lymphocyte antigen 4, ICAM1 intercellular adhesion molecule 1, LFA1 leukocyte function-associated antigen 1, PI3K phosphatidylinositol 3-kinase, SHP2 SRC homology 2-domain-containing protein tyrosine phosphatase 2, TCR T-cell receptor, ZAP70 ζ-chain-associated protein 70, LCK Src-family kinase

During T-cell development, the suppression of Nmt activity led to impaired TCR signals transmission, decreased T-cell activity, and cytokine secretion after the treatment of CpG and staphylococcal enterotoxin B (SEB) stimulation.^448^ In addition, lipids modification is common in lipid rafts, the structural components in living cell membrane. Lipid rafts are functional important in early T-cell receptor (TCR) signaling transduction. For example, the aggregation of lipid rafts is involved in TCR activation.^449,450^ After the presentation of an antigen by antigen-presenting cells (APC), T cells are activated through cell–cell contacts called immunological synapses (IS) or supramolecular activation cluster (SMAC).^72,451^ The SMAC can be divided into three concentric rings: the central SMAC, the peripheral SMAC, and the distal SMAC. Different SMAC parts enclose various signaling factors to form a stable immunological synapse for T-cell activation^452^ (Fig. 6). The central SMAC is enriched for TCR, MHC-peptide complexes and signaling molecules like PKC, Src-family kinase Lck and tyrosine kinase Fyn. The peripheral SMAC contains integrin-associated cytoskeletal proteins talin and lymphocyte function-associated antigen 1 (LFA1). The distal SMAC contains CD43, CD44, and CD45.^453–456^

Besides the centrosome translocation to the leading edge mediated by actin and microtubule networks, myristoylation makes a significant contribution to IS formation.^72,457^ The N-terminal glycine residue (glycine 2) is required for myristylation of tyrosine protein kinase Src family. Mutation of this residue blocked protein stability in the plasma membrane and inhibit TCR-induced tyrosine phosphorylation.^458^ Another Src-family member Fyn was observed for both N-myristylation and palmitylation, which altered its subcellular localization.^459^ More importantly, this dual-modification-mediated Fyn location in IS stabilizes the interactions between Fyn and TCRζ chain.^460^ Focal adhesion kinase (FAK) regulates cell adhesion to the ECM through integrin receptors and participates in integrin-mediated signal transductions, whose activity is influenced by myristoylated c-Src.^72,461^ N-myristoylation of Lck is indispensable for TCR signaling cascade including CD3ζ, Zap70, and ERK activation, as well as cytokines IFN-γ and IL-2 releasing.^448^ Cytoskeletal polarization and architecture organization are essential processes in T-cell activation to form F-actin-rich lamellipodium.^462^ F-actin organization is regulated by Arp2/3 complex to coordinate lamellipodia and filopodia transition in distinct cellular functions.^463^ The highly conserved Formin family proteins not only functions in filopodium formation, dynamic rearrangement of the actin cytoskeleton, and cell polarity but also is required in cytokinesis and endocytosis.^464^ The cytoskeletal regulation of Arp2/3 complex and Formins contributes in T-cell activation. Two kinds of Formins, mammalian diaphanous 1 (Dia1) and Formin-like-1 (FMNL1), displayed unique co-localization in the microtubule-organizing center (MTOC) and controlled TCR-mediated centrosome polarization.^464^ Importantly, N-terminal myristoylation plays essential roles in Formin’s membrane association, membrane trafficking, and bleb formation.^202,465^ These results support the function of protein myristylation in T-cell antigen receptor signaling.

#### Dendritic cells

Dendritic cells are efficient stimulators of B and T lymphocytes and mediate both innate and adaptive immune responses.^466^ DCs undergo maturation process to become highly activated. The metabolic function and/or metabolic byproducts in the tumor microenvironment are increasingly concerned and studied. Among these, the metabolism regulation of immune cells mediates cell differentiation, antigen presentation, ion signaling control, and cytoskeletal remodeling to facilitate downstream responses.^467^ NO is an essential metabolic regulator in monocyte-derived inflammatory DCs (moDCs) since its production inhibit mitochondrial oxygen consumption to change the OXPHOS and TCA cycle.^468^ NO synthase (NOS) enzymes include endothelial NOS (eNOS, NOS1) and neuronal NOS (nNOS, NOS3), which were reported to catalyze NO production.^469^ Based on the subcellular localization analysis of myristoylation-mutant eNOS cDNA, N-terminal myristoylation of the endothelial NO synthase was found to be functionally important in extracellular transport of highly cytotoxic products and signal transduction.^470^

The neddylation pathway in DCs is associated with its immune regulation function. The release of pro-inflammatory cytokines such as TNF-α and IL-6 by DCs is suppressed by neddylation inhibitor MLN4924 after the stimulation of LPS. The treatment caused a significantly reduced T-cell proliferation and interferon-γ secretion, without any change in apoptosis or cell viability. Mechanically, MLN4924 block the LPS-induced nuclear location of p65 NF-κB in BM-derived DCs (BMDCs) and prevent IκB degradation without affecting the MAPK/ERK pathway.^471^ IL-12p70 is a key cytokine produced by DCs, which is required to instruct an adaptive Th1 response.^472^ MLN9424 decreased the secretion of IL-12p70, indicating a restricted capacity of DCs for T-cell activation. Besides downregulated maturation and stimulatory capacity of DCs partially through the mTOR signaling pathway, MLN4924 also promoted caspase-dependent apoptosis of DCs and suppressed T-cell proliferation.^473^

#### T-cell function

T-cell activation and the corresponding immune response are along with cytoskeletal rearrangements by segregating engaged TCRs and promoting cytokines delivery.^462^ During T-cell polarization, MTOC is needed to translocate to IS, allowing T-cell secretion. The myristoylated actin nucleating Formins displayed MTOC-mediated migration without Arp2/3-dependent actin nucleation.^474^

Other kinds of PTM may affect T-cell activation, especially by metabolism regulation. The switch of T cell from quiescent states (naive and memory T cells) to highly proliferative states (developing thymocytes and effector T cells) is regulated by growth factor signals. One hallmark of this transition in metabolism is the change of ATP production from mitochondrial OXPHOS to high throughput glycolysis.^475^ The Warburg effect in tumor cells refers to the switch from TCA to aerobic glycolysis, leading to the production of lactate from glucose.^112^ The mitochondrial membrane-bound enzyme succinate dehydrogenase (SDH) functions in both the TCA cycle and electron transport chain.^476^ The inhibition of SDH led to decreased proliferation, neutralization of mitochondrial membrane, and impaired T-cell cytokine production, thus affect T-cell biology.^477^ Studies of Sirt5–SDH axis connect protein PTMs with cell metabolism, as well as T-cell function regulation. Indeed, the depletion of Sirt5 showed a substantial increase in SDH activity and enhanced SDH substrate succinate.^319^ Cytokines prevent proapoptotic factors release by regulating B-cell lymphoma 2 (BCL-2) activity to maintain mitochondrial integrity. In another way, growth factors promote nutrient uptake through activating protein kinases.^478^

The dramatically developed field in cancer therapy is immune checkpoint blockade. The immune system recognizes various antigens derived from outside pathogenic infection through TCRs, and is regulated by the immune checkpoint. Immune checkpoints are essential to prevent autoimmunity but often dysregulated in cancer development. The B7:CD28 family regulates co-stimulatory and inhibitory signals to keep a balance of T-cell activation and tolerance.^479^ The capacity for the selective recognition of antigens derived from any compartments in one cell, the capacity of CD8^+^ effector T cells (cytotoxic T lymphocytes (CTLs)) to recognize and kill antigen-expressing cells, and the capacity of CD4^+^ helper T cells to regulate immune responses make T cells a central role in cancer therapeutics.^480^ Cytotoxic T-lymphocyte-associated antigen 4 (CTLA4 or CD152) and programmed cell death protein 1 (PD-1 or CD279) are the most studied immunoinhibitory receptors. PD-1 ligand 1 (PD-L1, B7-H1 or CD274) and PDL2 (also B7-DC or CD273) are two ligands for PD-1.^481,482^ Their interaction may cause the inhibition of T-lymphocyte proliferation and cytokine production to suppress the immune response.^483^ High level of PD-L1 expression was reported in many types of cancer and myeloid cells in the tumor microenvironment.^484–489^

**Fig. 7:**
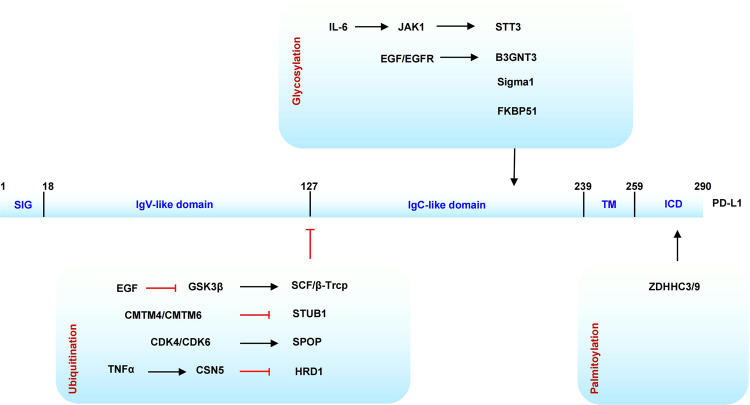
The modification of ubiquitination, palmitoylation, and glycosylation of the programmed cell death ligand 1 (PD-L1) protein

Studies revealed PD-L1-mediated immunosuppression can be regulated by several PTMs, indicating that except for expression regulation, targeting PTMs of PD-L1 may be a potential strategy to enhance antitumor immune responses (Fig. 7). As a multifunctional serine/threonine kinase, GSK3 regulates a large number of signal pathways through a phosphorylation cascade.^490^ In a recent study, PD-L1 was observed to undergo N-glycosylated at sites N35, N192, N200, and N219 to facilitate its stabilization. PD-L1 glycosylation antagonized its interaction with GSK3β, preventing GSK3β-mediated PD-L1 destabilization.^491^ In a study of triple-negative breast cancer, β-1,3-N-acetylglucosaminyltransferase (B3GNT3)-mediated PD-L1 glycosylation increased PD-L1 protein stability and PD-L1/PD-1 interaction. In the co-culture system of tumor cells with PBMC-derived activated T cells, the mutation of PD-L1 glycosylation sites exhibits more sensitivity to T cells.^492^ A spliced isoform of FK506-binding protein 51 (FKBP51s) catalyzed PD-L1 glycosylation to enhance its expression on the plasma membrane in glioblastoma.^493^ In CSCs, the EMT process induces N-glycosyltransferase STT3 expression through transcription factor β-catenin and produces a more stable N-glycosylated PD-L1.^494^ These results suggest that PD-L1 glycosylation is critical for PD-L1-mediated immunosuppression and targeting this PTM is an effective immune checkpoint therapy strategy.

#### Cytotoxic T cells

The inhibitors of farnesyltransferase and geranylgeranylation are reported to regulate immune cell proliferation, differentiation, and function. According to the types of cytokine production and distinct APC populations, the activated CD4^+^ T cells differentiate into three subtypes. (1) Th1 cells produce IFN-γ and lymphotoxin after the stimulate of cytokines IL-12 and IL-18 to regulate NFAT and NF-κB transcription factors, MAP kinase pathway, as well as the members of the IRF-1 family.^495^ (2) Th2 cells produce IL-4, IL-5, and IL-13 to regulate the expulsion of parasites and provide B-cell help.^495^ (3) Stimuli-like microbial factors, adjuvants including LPS or CpG, and β-glucan components in zymosan promote Th17 cells differentiation and production of IL-10, IL-17A, IL-17F, and IL-22 after TGF-β induction to regulate its anti-inflammatory fate in intestinal immune homeostasis maintenance.^496,497^ The CD4^+^CD25^+^Foxp3^+^ regulatory T cells (nTregs) function in the inhibition of naive T cells differentiate into pathogenic T-helper 1 effectors to suppress autoimmune disease and control a set of physiological and pathological immune responses.^498,499^ In addition, the depletion of isoprenoid intermediates reduced Ras farnesylation and RhoA geranylgeranylation, preventing Th1-mediated autoimmune disease experimental autoimmune encephalomyelitis (EAE).^500^ Simvastatin is a semisynthetic compound and cholesterol-lowering drug derived from lovastatin that inhibit HMG-CoA reductase activity. After TGF-β stimulation, simvastatin promotes Foxp3^+^ CD4^+^ T-cell differentiation. The same result was observed after GGTI-298 treatment but not FTI-277, suggesting protein geranylgeranylation but not protein farnesylation is responsible for regulating the balance between Th17 cells and Foxp3^+^ CD4^+^ T cells.^501^

Ca^2+^ channels (CRAC) in the plasma membrane is responsible for Ca^2+^ entry to mediate biological process as a second messenger in mast and T cells.^502^ The uptake and release of Ca^2+^ (also called Ca^2+^ handling) in mitochondrial regulate energy conversion, metabolism, differentiation, proliferation, gene expression, and immune response. The tumor suppressor Fus1, which is involved in Ca^2+^-myristoyl switch protein family, has been identified to balance mitochondrial changes during CD4^+^ T-cell activation.^503^ In addition, Fus1^−/−^ mouse exhibit abnormal inflammatory cytokine expression, altered T-cell differentiation and changed mitochondrial function.^504^ More importantly, the regulation function of Fus1 in Ca^2+^ handling depends on myristoylation. N-myristoylated Fus1 facilitates protein docking to the mitochondrial membrane to ensure mitochondrial Ca^2+^ handling and its tumor suppressor function.^505^ Cytotoxic T lymphocytes (CD8^+^ T cell, CTL) play an essential role in immune defense against intracellular pathogens like viruses, intracellular bacteria, and tumor. The interaction of Fas protein in target cells and Fas ligand (FaL) in CTLs activates caspase cascade in the targeted cells and induces apoptosis. The post-translational myristoylation of protein also regulates CTL function via its role in apoptosis.^72^ The activated caspase-8 cleaves the cytosolic form of proapoptotic molecule BID, resulting in a truncated fragment that relocates to mitochondria. The early study had identified N-myristoylation of BID after cleavage improved its insertion into mitochondria membrane to enhance cytochrome c release and cell death.^506^ Subsequently, many other proteins have been identified myristoylated and regulate nuclear fragmentation or cytoskeletal remodeling during apoptosis, these proteins include p21-activated kinase 2 (PAK2), gelsolin, cytoplasmic dynein intermediate chain 2 (DIC2 or CDIC2), B-cell receptor-associated protein 31 (Bap31), microtubule-actin cross-linking factor (MACF), and YTH domain family 2 protein (YTHDF2).^301^ The myristoylation occurs in caspase-cleaved PAK2 C-terminal kinase fragment to facilitate its location in membrane ruffles and internal membranes, resulting in c-JNK-dependent cell death.^507^ Targeting myristoylation-related protein subcellular location and cytoskeleton remodeling in apoptosis may lead to advancement in anticancer therapy.

#### PTMs in PD-L1 and functional regulation

T-cell-based immune response relies on the interaction of TCR on T cells with peptide-major histocompatibility complexes (MHC) on target cells. A series of spatiotemporal control by co-stimulatory or co-inhibitory receptors and their ligands (immune checkpoints) contributes to T-cell activation and function.^508^ Recently, novel immune checkpoint molecules have been intensively studied except for PD-L1 and CTLA4. These targets contain B- and T-lymphocyte attenuator (BTLA), lymphocyte activation gene-3 (LAG-3), T-cell immunoglobulin and mucin-domain-containing-3 (TIM-3), T-cell immunoglobulin and ITIM domain (TIGIT), and VISTA.^509^ PD-1 (CD279) is a receptor of the Ig superfamily and can be expressed CD4^+^ and CD8^+^ T cells, B cells, macrophages, natural killer T (NKT) cells, and some subset of dendritic cells (DCs) after the immune stimulus.^509^ Two ligands PD-L1 (CD274, B7-H1) and PDL2 (CD273, B7-DC) bind PD-1 to inhibit T‐cell activation and proliferation, resulting in maintenance of homeostasis and prevention from autoimmunity. PD‐L1 is expressed on several types of cancer as well as on tumor-infiltrating immune cells in the tumor microenvironment. Several signal pathways (MAPK, MEK-ERK pathway and the phosphoinositide 3‐kinase (PI3K)/Akt pathway), the transcription factors (MYC, AP-1, HIF-1, signal transducer, and activator of transcription 3 (STAT3), NF‐ĸB, TGF‐β, GATA‐3, and T‐box transcription factor T‐bet), as well as the activation of KRAS, EGFR, and anaplastic lymphoma kinase (ALK) regulate PD-L1 expression.^510,511^ PD-1 and PD-L1 interaction not only results in self-tolerance activation but also provides a strategy to escape immune surveillance.^512^ Considering the central role of PD-L1 in the fundamental biology of immune escape and its predictive value for clinical response, a deep understanding of PD-L1 regulation is need to design effective cancer immune checkpoint therapies. The complex regulatory network of PD-L1 level includes (1) genetic alterations at the PD-L1 locus, (2) inflammatory signals such as pro-inflammatory cytokine IFN-γ and type I interferons (IFN-α and IFN-β), (3) aberrant oncogenic signaling pathways, (4) microRNA-mediated PD-L1 mRNA regulation, and (5) post-translational modulation.^511^ PD-L1 expression, stability, and the activity to bind PD-1 can be directly regulated by several proteins. With a whole-genome CRISPR/Cas9 deletion library screen, CKLF-like MARVEL transmembrane domain-containing protein 6 (CMTM6) was identified as a major regulator of PD-L1 expression. CMTM6 colocalized with PD-L1 at the plasma membrane and in recycling endosomes to reduce its ubiquitination and prevents PD-L1 from lysosome-mediated degradation. Meanwhile, CMTM6 also fulfills a similar role in the immunological synapse between T cells and cancer cells or APCs.^513^ Another family member CMTM4 was responsible for the positive regulation of PD-L1 expression in CMTM6 deficient cells.^514^ The cyclin-dependent kinase 4 (CDK4) phosphorylates SPOP, the E3-ubiquitin ligase of PD-L1, and stabilizes SPOP by dissociating it from the ubiquitin E3-ligase complex APC/C^Cdh1^. The evaluated SPOP promotes ubiquitination-mediated PD-L1 degradation, leading to decreased PD-L1 level.^515^

In addition to ubiquitination, other protein PTMs of PD-L1 also modulate its immunosuppression activity. The serine/threonine protein kinase GSK3β binds to and phosphorylates PD-L1, leading to ubiquitination-associated PD-L1 degradation. This process was blocked by EGF signal-induced PD-L1 glycosylation.^491^ Moreover, glycosylation of PD-L1 is necessary for PD-L1 and PD-1 interaction. A type II transmembrane protein B3GNT3 binds to and catalyzes PD-L1 N-linked glycosylation and B3GNT3 depletion inhibit tumor cell growth.^492^ COP9 signalosome (CSN) specific phosphorylation can target substrates to ubiquitin-dependent degradation.^516^ One of the family members CSN5 also possesses deubiquitination activity and positively regulates PD-L1. NF-kB induced CSN5 expression, resulting in CSN5-dependent PD-L1 deubiquitination and stabilization.^511,517^ In the current studies, palmitoylation was discovered on PD-L1 and played an important role in protein stability. PD-L1 expression was decreased after palmitoylation inhibitor treatment. The palmitoylation of PD-L1 also protected cancer cells from T-cell killing and promoted tumor growth. The cysteine-rich zinc finger protein family member ZDHHC9 is responsible for PD-L1 palmitoylation to maintain its stability and cell surface distribution.^518^ In addition, PD-L1 palmitoylation also blocks its ubiquitination and lysosome-associated degradation, with ZDHHC3 as the main acetyltransferase. Inhibitors targeting PD-L1 palmitoylation were proved to be effective to overcome PD-L1-mediated immune evasion.^519^ Anti-PD-1/PD-L1 agents and combination therapies with hundreds of chemotherapies, radiotherapy, and anti-angiogenic agents have been tested in active clinical trials and serve as promising attempt in the cancer immunotherapy field.^520^ Targeting the novel interchange between various PTMs of PD-L1 protein offer novel opportunities to combat immune surveillance and develop more effective drugs and treatments from a new perspective.

## Therapy targets

Protein PTMs play key roles in the regulation of cellular hemostasis by targeting both histones and non-histones. Over the past few years, HDAC inhibitors have been discovered and reviewed both as single anticancer agents and in combination with other chemotherapeutic drugs/radiotherapy.^521–531^ For example, the histone deacetylase inhibitor suberoylanilide hydroxamic acid (SAHA; vorinostat (Zolinza)) may cause cell death in tumor cells and tumor-bearing animals. SAHA has been approved by the US Food and Drug Administration for clinical trials.^532^ Another potent histone deacetylase inhibitor romidepsin induces cell-cycle arrest or selective apoptosis of malignant cells and has been recently FDA approved in several cancer types, including solid tumors and hematologic malignancies.^533,534^

Considering the oncogenic function of Ras proteins and their malignant transformation regulation by farnesylation, strategies targeting this PTM are processed to treat cancer a long time ago. Many compounds were identified by random screening to block Ras farnesylation or farnesyl-protein transferase (FPTase) in RAS-transformed cells, human cancer cells, and animal models with no toxicity to normal cells or to normal tissues.^535^ The anticancer function of farnesyltransferase inhibitors and GGTase I inhibitors on cell-cycle control to induce apoptosis and the related bench-to-bedside translational strategies have been reviewed in detail.^536–538^ Inhibitors of farnesyltransferase are developed to block farnesyl attachment such as the benzodiazepine peptidomimetics, the CA1A2X peptidomimetics family, and the non-peptide mimetics of CAAX.^539–542^ The inhibition of isoprenoid biosynthesis and farnesylation reaction are strategies less direct than the inhibition of FPTase to inhibit Ras farnesylation. Targeted screens and rational designs based on substrates are developed to find proper inhibitors.^543–546^ The CAAX peptidomimetics L-739,749 inhibited anchorage-independent growth in RAS-transformed Ratl cells and was also effective in animal models.^547^ Other competitive inhibitors targeting farnesyl diphosphate (FPP) were designed and identified for inhibition of Ras processing in NIH3T3 fibroblasts.^544,548^ K-Ras was reported to show a higher affinity for farnesyltransferase than H-Ras as well as the substrate potential for geranylgeranyltransferase-1. The inhibitor BZA-2B caused the suppression effect of K-RasB both in its farnesylation and geranylgeranylation activity.^549^ Moreover, the inhibitor L-744,832 was observed repressed tumor growth in both (MMTV)-v-Ha-RAS transgenic mice and MMTV-N-RAS mice.^550,551^ Several preclinical studies of farnesyltransferase displayed antitumor effect by blocking cell proliferation, inducing apoptosis, increasing reactive oxygen species and causing DNA double-strand breaks with single inhibitors, dual inhibitors, and combination treatments with chemotherapies.^552^

For the clinical study, an orally bioavailable, nonpeptidomimetic methylquinolone farnesyltransferase inhibitor tipifarnib (Zarnestra, R115777) has exhibited effectiveness in patients with high-risk myelodysplasia, myeloproliferative disorders, and imatinib-resistant chronic myelogenous leukemia.^553^ In human breast cancer in vivo, tipifarnib inhibits FTase activity and is associated with reduced phospho (p)-STAT3.^554^ Despite these studies, hundreds of proteins undergo farnesylation, making the mechanisms of drug resistance and several activated pro-survival pathways necessary to be illustrated. In fact, the combination strategy of FTase inhibitors with group I PAK inhibitors is effective in human melanoma, lung, and colon cancer.^555^ Another farnesyltransferase inhibitor lonafarnib exhibit sufficiently melanoma cell growth inhibition and apoptosis induction after the combination with the pan-RAF inhibitor sorafenib.^556^ Besides tipifarnib and lonafarnib, other inhibitors including lestaurinib, tandutinib, and PKC 412 have also been designed and tested under early-phase clinical evaluation in acute myeloid leukemia (AML).^557^ The ongoing clinical trials are listed in Table 2.

**Table 2:** The ongoing clinical trials of Farnesyl-transferase inhibitors

NCT number	Conditions	Drug	Phases
NCT02535650	Urothelial carcinoma	Tipifarnib	Phase 2
NCT02210858	Chronic myeloid leukemia	Tipifarnib	Phase 1|Phase 2
NCT00847223	Mantle cell lymphoma	Tipifarnib	Phase 2
NCT00612651	Gliosarcoma	Temodar and SCH66336	Phase 1
NCT00354146	Leukemia, nonlymphocytic, acute	Tipifarnib (R115777)	Phase 2
NCT00112853	Leukemia	Tipifarnib|Etoposide	Phase 1
NCT00102635	Head and neck cancer	Fenretinide (4-HPR) | Drug: SCH66336	Phase 1
NCT00101153	Leukemia	Cytarabine|Daunorubicin hydrochloride|Tipifarnib	Phase 1
NCT00096122	Leukemia	Cytarabine|Idarubicin|Tipifarnib	Phase 1|Phase 2
NCT00093990	Leukemia	Tipifarnib;Zarnestra; R115777	Phase 3
NCT00093470	Leukemia	Tipifarnib	Phase 3
NCT00093418	Leukemia	Tipifarnib	Phase 2
NCT00004009	Leukemia	Tipifarnib	Phase 1
NCT00005041	Lung cancer	Tipifarnib	Phase 2
NCT00012350	Multiple myeloma	FTI	Phase 2
NCT00083096	Brain and central nervous system tumors	lonafarnib|Temozolomide	Phase 1
NCT00005989	Lung cancer	Tipifarnib	Phase 2
NCT00020774	Liver cancer	Gemcitabine hydrochloride|lonafarnib	Phase 2
NCT00006376	Bladder cancer|transitional cell cancer of the renal pelvis and ureter	Chemotherapy|Tipifarnib	Phase 2
NCT00006085	Unspecified adult solid tumor, protocol specific	CP-609,754	Phase 1
NCT00005848	Prostate cancer	Chemotherapy|Tipifarnib	Phase 2
NCT00005843	Pancreatic cancer	Tipifarnib	Phase 2
NCT00077519	Pancreatic cancer	Tipifarnib|Radiation	Phase 1
NCT00077363	Recurrent breast cancer|stage iv breast cancer	Tipifarnib|Capecitabine	Phase 2
NCT00076102	Neurofibromatosis 1|neurofibroma, plexiform	Pirfenidone	Phase 2
NCT00006213	Leukemia	BMS-214662	Phase 1
NCT00073450	Carcinoma, squamous cell|head and neck neoplasms	Farnesyl-protein transferase inhibitor	Phase 2
NCT00070252	Adult solid neoplasm| breast carcinoma	Capecitabine|Docetaxel|Tipifarnib	Phase 1|Phase 2
NCT00005845	Leukemia	Tipifarnib	Phase 1
NCT00003707	Unspecified adult solid tumor, protocol specific	Gemcitabine hydrochloride|Tipifarnib	Phase 1
NCT00022529	Unspecified adult solid tumor, protocol specific	BMS-214662|Trastuzumab|Pharmacological study	Phase 1
NCT00006242	Unspecified adult solid tumor, protocol specific	BMS-214662	Phase 1
NCT00055757	Stage IIIB non-small cell lung cancer|stage IV non-small cell lung cancer	Tipifarnib|Cisplatin|Gemcitabine hydrochloride	Phase 2
NCT00054470	Breast cancer	Trastuzumab|Tipifarnib	Phase 2
NCT00045396	Leukemia	Tipifarnib	Phase 2
NCT00027872	Leukemia	Tipifarnib	Phase 2
NCT00026104	Adenocarcinoma of the pancreas| pancreatic cancer	Gemcitabine hydrochloride|Paclitaxel|Tipifarnib|Radiation	Phase 2
NCT00025480	Lung cancer	Carboplatin|Paclitaxel|Tipifarnib|Radiation	Phase 1
NCT00025038	Juvenile myelomonocytic leukemia	Tipifarnib|Isotretinoin|Fludarabine phosphate|Cytarabine|Radiation|Cyclophosphamide|Anti-thymocyte globulin	Phase 2
NCT00006351	Bladder cancer|transitional cell cancer of the renal pelvis and ureter|urethral cancer	Gemcitabine hydrochloride|lonafarnib	Phase 2
NCT00005990	Unspecified adult solid tumor, protocol specific	Tipifarnib|Topotecan hydrochloride	Phase 1
NCT00005973	Unspecified adult solid tumor, protocol specific|unspecified childhood solid tumor, protocol specific	BMS-214662|Other: laboratory biomarker analysis	Phase 1
NCT00050986	Glioblastoma multiforme	Temozolomide|R115777	Phase 1|Phase 2
NCT00050336	Carcinoma, non-small-cell lung|metastases, neoplasm	Lonafarnib (SARASAR)	Phase 3
NCT00050141	Breast cancer	ZARNESTRA, Tipifarnib, R115777	Phase 2
NCT00048503	Acute myeloid leukemia	ZARNESTRA, Tipifarnib, R115777	Phase 2
NCT00040547	Neoplasms	Farnesyl-Protein Transferase Inhibitor	Phase 1
NCT00040534	Neoplasms	Farnesyl-Protein Transferase Inhibitor	Phase 1
NCT00038597	Myelogenous leukemia, chronic	SCH66336	Phase 2
NCT00038493	Glioblastoma multiforme	Temozolomide and SCH66336	Phase 2
NCT00034684	Leukemia	Farnesyl-protein transferase inhibitor	Phase 1|Phase 2
NCT00021541	Neurofibroma, plexiform|neurofibromatosis type I	Tipifarnib|Other: placebo	Phase 2

Components targeting the ubiquitin–proteasome system is an effective strategy for the treatment of cancer. MLN4924 is a small-molecule inhibitor of NAE screened through a high throughput assay. MLN4924 treatment resulted in S-phase disruption and suppressed tumor growth both in vivo and in vitro.^558^ Pharmaceutically, MLN4924 forms an irreversible covalent adduct with NEDD8 to disrupt the thioester bond between NEDD8 with NAE and block substrates neddylation.^559^ The In eukaryotes, ORC (origin recognizing complex) recognizes and binds to replication origins to initiate DNA replication, following recruitment of replication licensing factors Cdt1 and Cdc6, as well as MCM complex to form pre-replicative complex (pre-RC).^560–562^ The MLN4924 treatment also increased and stabilized Cdt1 protein level, leading to DNA re-replication, the activation of apoptosis, and senescence. Interestingly, compared to wide type cells, p53^−/−^ and p21^−/−^ cells appeared to be more sensitive to MLN4924.^563^ In addition to S-phase arrest and appearance of re-replicated DNA, MLN4924 was reported to cause other phenotypes including Cullin-RING ligase (CRL) substrates degradation and ATM/ATR kinase-associated single-strand and double-strand breaks.^564^ Based on these results, MLN4924 has been studied as a promising and effective anticancer strategy. Several clinical trials of MLN4924 are ongoing in anticancer therapy. For the treatment of AML, MLN4924 dramatically disrupted AML cells growth, inhibit clone formation, and decreased Cullin-dependent substrates expression, causing the inhibition of NF-κB transcriptional activity and increased reactive oxygen species (ROS) generation.^565^ However, there are different biological mechanisms of MLN4924 in distinct genetic subgroups of diffuse large B-cell lymphoma (DLBCL). MLN4924 downregulated NF-κB target genes in activated B-cell-like (ABC) DLBCL and inducted DNA re-replication in germinal-center B-cell-like (GCB) DLBCL.^566^ The combination strategy of NAE inhibitors showed potential benefit. MLN4924 treatment increased radiosensitivity in pancreatic cancer cells with enhanced G2/M arrest, aneuploidy, and apoptosis. Mechanistically, the inhibition of Cullin neddylation by MLN4924 increased the accumulation of SCF (SKP1, Cullins, and F-box protein) E3 substrates CDT1 and WEE1 after radiation, followed by a remarkable increase in DNA damage and genomic instability.^567^ Cisplatin is one of the widely used anticancer agents by the formation of inter-strand DNA cross-linkages (ICL) to damage DNA, inhibit DNA synthesis and induce tumor cell death.^568^ Fanconi anemia (FA) pathway is responsible for resolving ICLs to maintain genomic stability, in which FANCD2 mono-ubiquitination is a central event.^569^ MLN4924 can suppress ICL-induced FANCD2 mono-ubiquitination, enhancing sensitivity to DNA cross-linking agents.^570^ In addition, MLN4924 functions not only in the induction of apoptosis to suppress cancer cell growth but also in the induction of an irreversible senescence through persistent accumulation of p21 and sustained activation of DNA damage response.^571^ In response to the multiunit CRL inactivation, protective autophagy was induced by MLN4924. The corresponding phenotypes of inhibition of the mTOR pathway, accumulation of DEPTOR and HIF-1α, and ROS-induced stress were observed.^572,573^ More recently, MLN4924 has been shown to suppress cell migration, proliferation, and tube formation in vitro, as well as angiogenesis and tumorigenesis in vivo.^574^ In conclusion, inhibitors targeting neddylation like MLN4924 may have potential as chemotherapeutics. The clinical trials of MLN4924 are listed in Table S1. However, cancer cells with mutations in the NAE subunit UBA3 have developed MLN4924 resistance and restored cell survival.^575,576^ Other small-molecule inhibitors are discovered to target E2 conjugating enzyme (UBE2M or UBC12) and DCN1 (DCUN1D1) interaction and DCN1-mediated Cullin neddylation.^577,578^ The treatment of compound WS-383 inhibited Cul3/1 neddylation, leading to the accumulation of p21, p27, and erythroid 2-related factor 2 (NRF2).^579^ High-affinity inhibitors DI-404 and DI-591 bind to cellular DCN1 and DCN2 and disrupt the association of DCN1 and UBC12, selectively inhibiting Cullin-3 neddylation and increased NRF2 level.^580,581^

Given that NMT-mediated protein myristoylation participates widely in signaling processes in many types of cancer, NMT can be considered as a potential target for cancer treatments. The novel ceramidase inhibitors (1 S,2 R)-N-myristoylamino-phenylpropanol-1 (d–e-MAPP) and (1 R,2 R)-N-myristoylamino-4′-nitro-phenylpropandiol-1,3 (B13) was synthesized and examined to block Src N-myristoylation.^582^ As with NMT1 knockdown, a small-molecule inhibitor B13 blocked NMT1 enzymatic activity and suppressed proliferation, migration, and invasion in vitro and tumor growth in vivo.^260^ Tris (dibenzylideneacetone) dipalladium (Tris DBA), an organopalladium compound identified in the melanoma study, showed NMT1 inhibition function. This compound was found to have the significant activity to block tumor growth through decreased downstream signaling pathways, including MAPK kinase, PI3K, and Stat-3.^583^ For the sake of drug resistance of tyrosine kinase inhibitors (TKIs) harboring mutations in the BCR-ABL kinase in chronic myelogenous leukemia (CML), several inhibitors like asciminib (ABL001) and GNF-2 were discovered. Those inhibitors occupied the internal myristate binding site/pocket in the ABL kinase domain to recover the negative regulatory effect of TKIs.^584–587^ A series of phase I studies are ongoing (ABL001 + Nilotinib, ABL001 + Imatinib, and ABL001 + Dasatinib) in CML or Ph+ ALL patients (https://clinicaltrials.gov/ct2/show/NCT02081378).

### Concluding remarks and perspectives

This review highlights the fundamental questions of several highly preserved but less-studied protein PTMs, including the basic signature, the techniques or predictive tools to detect these PTMs, the function and regulation in cancer biology, and suppression effects in the tumor microenvironment. Further characterization of the functional impacts of these novel PTMs on immune regulation may extend our understanding of the multifunctional regulatory network of TME and provide new ideas to improving immunotherapy efficacy.

Given the numerous and diverse links of tumor cell and TME regulation, drugs targeting PTMs have proven rather effective both in preclinical and clinical studies. However, numerous hurdles and uncertainties remain in targeting PTMs into clinical practice. The key challenges include: (1) how does metabolic associated PTMs regulate innate and acquired immunity in cancer progression and treatment; (2) that it is hard to predict a specific modification and its effect in compensatory cellular pathways considering the heterogeneity and plasticity of TME; (3) that it is necessary to identify specific drug targets and biological mechanisms after the treatment of PTM inhibitors; (4) that the effects of endogenous factors, such as patients’ clinical features and microbiota in TME, on the outcomes of treatments remain to be determined; (5) that it is a hot area to construct a preclinical model of TME regulation based on different PTMs and their crosstalk; (6) that an increased understanding of the dominant drivers of PTM regulation is needed to enable reprogramming of TME, which is available for tumor elimination; and (7) that an effective and efficient assessment of PTMs regulation in combination with TME shaping in early-phase clinical studies has the potential to improve the cost-effectiveness of anticancer treatment.

Addressing these challenges will require the combined efforts of basic researchers and clinicians, and the focusing of resources to accelerate understanding of the complex interactions between PTMs regulation and the TME shaping system and the development of improved treatment options for patients with cancer.

## Supplementary Information

Supplementary Information
