# Correction: Insights into the post-translational modification and its emerging role in shaping the tumor microenvironment

**DOI:** 10.1038/s41392-022-00901-7

**Published:** 2022-01-31

**Authors:** Wen Li, Feifei Li, Xia Zhang, Hui-kuan Lin, Chuan Xu

**Affiliations:** 1 Integrative Cancer Center & Cancer Clinical Research Center, Sichuan Cancer Hospital & Institute, Sichuan Cancer Center, School of Medicine, University of Electronic Science and Technology of China, 610042 Chengdu, P. R. China; 2 Guangxi Collaborative Innovation Center for Biomedicine (Guangxi-ASEAN Collaborative Innovation Center for Major Disease Prevention and Treatment), Guangxi Medical University, 530021 Nanning, Guangxi China; 3 Institute of Pathology and Southwest Cancer Center, Southwest Hospital, Third Military Medical University (Army Medical University), 400038 Chongqing, China; 4 Department of Cancer Biology, Wake Forest Baptist Medical Center, Wake Forest University, Winston Salem, NC 27101 USA

**Keywords:** Cancer microenvironment, Oncogenes

Correction to: *Signal Transduction and Targeted Therapy* 10.1038/s41392-021-00825-8, published online 20 December 2021

After online publication of the article^1^, the authors noticed some of the chemical structures in Fig. 1 are nonstandard forms that need to be corrected. The correct figure is provided as follows. At the same time, an author’s name is incorrectly written. The author of “Huikuan Lin” should be “Hui-Kuan Lin”. We also noticed a mistake in the sentence “The covalent attachment of glycans to proteins and lipids is called protein glycosylation, which is a common contribution to structure and modification diversity in eukaryotes” in the “O-GlcNAcylation” section of page 6 line 45-47, which should be “The covalent attachment of glycans to proteins is called protein glycosylation, which is a common contribution to structure and modification diversity”. The key findings of the article are not affected by these corrections.

The original article has been corrected.

**Fig. 1:**
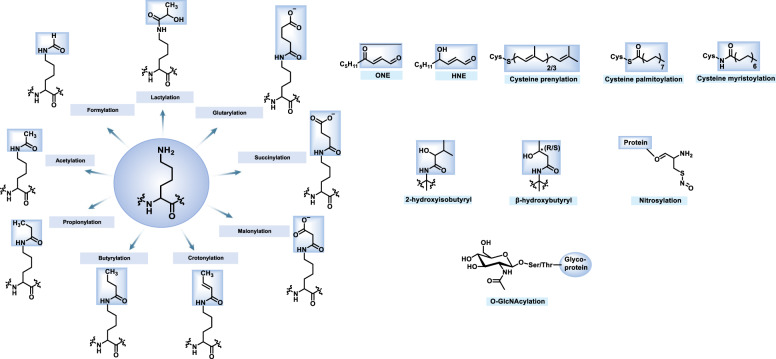
Chemical structures of histone/non-histone PTMs in this review
