# Supplementary material for: Insights into the post-translational modification and its emerging role in shaping the tumor microenvironment
Source: Signal Transduct Target Ther. 2021 Dec 20;6:422. doi: 10.1038/s41392-021-00825-8 (PMC8685280; doi:10.1038/s41392-021-00825-8)
Supplement: Supplementary file 1 — Supplementary Information [file 41392_2021_825_MOESM1_ESM.docx]

Table 3. Clinical trials of MLN4924 (Pevonedistat)

| **NCT Number** | **Conditions** | **Phases** |
| --- | --- | --- |
| NCT01814826 | Acute Myelogenous Leukemia (AML) | Phase 1 |
| NCT03772925 | Recurrent or refractory AML \| Recurrent or refractory Myelodysplastic Syndrome | Phase 1 |
| NCT01011530 | Metastatic Melanoma | Phase 1 |
| NCT03770260 | Recurrent Plasma Cell Myeloma \| Refractory Plasma Cell Myeloma | Phase 1 |
| NCT01862328 | Solid Tumors | Phase 1 |
| NCT03330106 | Advanced Solid Neoplasm | Phase 1 |
| NCT03965689 | Lung Cancer | Phase 2 |
| NCT00677170 | Advanced Nonhematologic Malignancies | Phase 1 |
| NCT03813147 | Acute Myeloid Leukemia | Phase 1 |
| NCT02122770 | Advanced Solid Tumors | Phase 1 |
| NCT03323034 | Lymphoma \| Malignant Solid Neoplasm \| Primary Central Nervous System Neoplasm | Phase 1 |
| NCT03459859 | AML \| Advanced Myelodysplastic Syndromes \| MDS | Phase 1 |
| NCT03057366 | Advanced Solid Tumors, Neoplasms, Advanced Solid | Phase 1 |
| NCT00722488 | Hematologic Malignancies \| Multiple Myeloma \| Lymphoma \| Hodgkin Lymphoma | Phase 1 |
| NCT03486314 | Advanced Solid Neoplasm | Phase 1 |
| NCT00911066 | AML \| Acute Lymphoblastic Leukemia \| Myelodysplastic Syndrome | Phase 1 |
| NCT04800627 | Locally advanced, metastatic and unresectable Malignant Solid Neoplasm | Phase 1\|Phase 2 |
| NCT03745352 | Recurrent Acute Myeloid Leukemia \| Refractory Acute Myeloid Leukemia | Phase 2 |
| NCT03386214 | Myelofibrosis | Phase 1 |
| NCT04266795 | Acute Myeloid Leukemia (AML) | Phase 2 |
| NCT03814005 | Myelodysplastic Syndromes \| Leukemia \| Renal Insufficiency \| Liver Disease \| Neoplasms | Phase 1 |
| NCT03709576 | Acute Myeloid Leukemia (AML) | Phase 2 |
| NCT01415765 | Lymphoma | Phase 1\|Phase 2 |
| NCT03479268 | Leukemia \| Lymphoma \| Richter Syndrome | Phase 1 |
| NCT03009240 | Acute Myeloid Leukemia | Phase 1 |
| NCT03330821 | Acute Myeloid Leukemia | Phase 1\|Phase 2 |
| NCT02782468 | Leukemia \|Myelodysplastic Syndromes | Phase 1 |
| NCT04985656 | Myelodysplastic Syndromes (MDS) | Phase 2 |
| NCT04175912 | Metastatic Cholangiocarcinoma \| Hepatocellular Carcinoma | Phase 2 |
| NCT03268954 | Myelodysplastic Syndrome \| Leukemia | Phase 3 |
| NCT02610777 | Myelodysplastic Syndrome \| Leukemia | Phase 2 |
| NCT03862157 | Leukemia \| Essential Thrombocythemia \| Myelodysplastic Syndrome | Phase 1\|Phase 2 |
| NCT03319537 | Mesothelioma | Phase 1\|Phase 2 |
| NCT04090736 | Leukemia, Myeloid, Acute | Phase 3 |
| NCT04712942 | AML in Remission \| Myelodysplastic Syndrome \| Minimal Residual Disease | Phase 2 |
| NCT04172844 | Acute Myelogenous Leukemia | Phase 1 |
| NCT03228186 | Non-small Cell Lung Cancer | Phase 2 |
| NCT04484363 | Myelodysplastic Syndromes |  |
| NCT03349281 | Refractory and relapsed Acute Lymphoblastic Leukemia | Phase 1 |
| NCT03238248 | Myelodysplastic Syndrome \| Myeloproliferative Neoplasm | Phase 2 |
| NCT03013998 | Previously Untreated Acute Myeloid Leukemia | Phase 1\|Phase 2 |
